# Bacterial Composition and Survival on Sahara Dust Particles Transported to the European Alps

**DOI:** 10.3389/fmicb.2015.01454

**Published:** 2015-12-22

**Authors:** Marco Meola, Anna Lazzaro, Josef Zeyer

**Affiliations:** Environmental Microbiology, Institute of Biogeochemistry and Pollutant Dynamics, Environmental Systems Science, Swiss Federal Institute of TechnologyETH Zurich, Zurich, Switzerland

**Keywords:** Saharan dust event, Jungfraujoch, snow, bioaerosols, MiSeq Illumina, Biolog, UV radiation, desiccation stress

## Abstract

Deposition of Sahara dust (SD) particles is a frequent phenomenon in Europe, but little is known about the viability and composition of the bacterial community transported with SD. The goal of this study was to characterize SD-associated bacteria transported to the European Alps, deposited and entrapped in snow. During two distinct events in February and May 2014, SD particles were deposited and promptly covered by falling snow, thus preserving them in distinct ochre layers within the snowpack. In June 2014, we collected samples at different depths from a snow profile at the Jungfraujoch (Swiss Alps; 3621 m a.s.l.). After filtration, we performed various microbiological and physicochemical analyses of the snow and dust particles therein that originated in Algeria. Our results show that bacteria survive and are metabolically active after the transport to the European Alps. Using high throughput sequencing, we observed distinct differences in bacterial community composition and structure in SD-layers as compared to clean snow layers. Sporulating bacteria were not enriched in the SD-layers; however, phyla with low abundance such as *Gemmatimonadetes* and *Deinococcus-Thermus* appeared to be specific bio-indicators for SD. Since many members of these phyla are known to be adapted to arid oligotrophic environments and UV radiation, they are well suited to survive the harsh conditions of long-range airborne transport.

## Introduction

Aerosols are suspended liquid, solid or multiple-phase particles of condensed matter in a gaseous medium. They vary in composition (i.e., biological or mineral), size (0.001–100 μm) and shape (Kulkarni et al., 2011). In the past few years, aerosols have received increasing attention as important agents of climate change (Solomon et al., 2011).

Arid regions such as deserts are the major source of mineral dust particles in the atmosphere. There, dust particles are uplifted by storm activity and transported as aerosols to altitudes above 5000 meters above sea level (m a.s.l.) (Prospero et al., 2005). The Sahara Desert in Africa is the world's main source of mineral aerosols (Goudie and Middleton, 2006) and has been estimated to contribute 630–710 Mt to the atmosphere each year (D'Almeida, 1986). Approximately 60% of the Saharan dust emissions are transported southward, 25% westward to the Atlantic, 5% eastward to the Middle East and 10% northward to Europe (Shao et al., 2011).

Up to 25% of the total global aerosol mass is composed of biological particles, or bioaerosols (Jaenicke, 2005). The first descriptions of bioaerosols were provided by Charles Darwin, who observed “67 different organic forms” in fine dust particles that were deposited on the *Beagle* during his journey across the Atlantic Ocean in 1833 (Darwin, 1846). Generally, bioaerosols contain skin fragments, fur fibers, protein crystals, pollen, plant fragments, spores, viruses, algae, fungi, and bacteria, and can be free-floating or attached to mineral aerosols (Després et al., 2012; Deleon-Rodriguez et al., 2013). Bioaerosols such as viruses, bacteria and fungi have gained increasing interest because of their potential to spread pathogens over long distances (Prospero et al., 2005; Griffin, 2007; Polymenakou, 2012). They have also been linked with atmospheric processes (Morris et al., 2011; Šantl-Temkiv et al., 2013). For example, airborne bacteria can serve as ice-nuclei during snow precipitation (Amato et al., 2007b; Bowers et al., 2009; Hiranuma et al., 2015).

The long-distance transport of viable bacteria in conjunction with mineral aerosols may represent a way for bacteria to colonize new environments and contribute to an increase in diversity at remote terrestrial and aquatic habitats (Barton et al., 2010). In fact, airborne bacteria from Sahara dust (SD) particles collected on Darwin's *Beagle* in the Nineteenth century were shown to be viable 150 years later (Gorbushina et al., 2007). However, the extent to which the organisms remain viable or active after the aerial dispersal are still uncertain, as well as their potential to establish a colony after deposition (Pointing and Belnap, 2012). Due to the harsh conditions associated with airborne transport (e.g., desiccation stress, UV exposure, oligotrophic conditions, and low temperatures) (Smith et al., 2009), only specially adapted taxa are able to survive the journey to a new environment. Sporulation or pigmentation might help to protect bacteria against the harsh conditions that prevail during transport (Tong and Lighthart, 1997). For example, *Janthinobacterium* is a bacterium that is commonly detected in air samples and produces an indigo-purple pigment (Fahlgren et al., 2010).

To date, airborne bacteria from the Sahara Desert have been sampled along the coast of Greece and southeastern Mediterranean (Griffin et al., 2007; Polymenakou et al., 2008; Katra et al., 2014), in Spain (Sánchez De La Campa et al., 2013; Barberán et al., 2014) and in the Caribbean (Griffin et al., 2003; Prospero et al., 2005). Air sampling of bacteria can be performed using techniques such as filtration, impaction, suction, impingement and electrostatic precipitation, each of which presents different advantages and disadvantages for specific downstream biological analyses (e.g., PCR-based techniques) (Mandal and Brandl, 2011). The collection of aerosols, either indoors or outdoors, requires a understanding of the physical principles influencing the collection of suspended particles that can cause quantitative and qualitative biases (Després et al., 2012). Notably, the collection of bioaerosols requires special handling procedures to avoid damaging the cells during the collection process (e.g., dehydration stress) (Després et al., 2012). Finally, the timing and duration of Saharan dust events (SDEs) remains difficult to predict, hampering the ability of researchers to recover an air volume suitable for microbiological analyses.

Bacteria deposited on snow allow researchers to circumvent the drawbacks of air sampling. Both the physical integrity and the potential viability of the bacterial cells are preserved, as they are collected at freezing temperatures from within the snowpack. High-altitude locations above the influence of the planetary boundary layer are ideal for detecting deposited Sahara dust (SD) particles because the potential contamination from anthropogenically-derived aerosols after deposition is minimal (Griffiths et al., 2014; Nicolás et al., 2014).

Most SDE studies performed in the Alps have focused on mineralogical and chemical aspects (Sodemann et al., 2006; Thevenon et al., 2009); microbiological analyses are still lacking. The first attempts in this direction were made by González-Toril et al. (2009) and Chuvochina et al. (2011a) on the Mont Blanc glacier. However, no details regarding the physicochemical parameters of the snow, the position of the Sahara dust layers (SD-layers) within the snowpack or the geochemical characteristics of the dust particles were presented in these studies. Despite the valuable contribution of these preliminary studies, the low sequencing depth achieved by small clone libraries makes it difficult to draw solid conclusions about the bacterial community structures associated with SDEs. Moreover, knowledge is still limited regarding the viability and metabolic activity of the bacteria transported from the Sahara Desert to the Alps.

The region of Jungfraujoch, located in the Swiss Alps at about 3600 m a.s.l., lies above the influence of the planetary boundary layer and is therefore a highly advantageous location for studying aerosols transported from the Sahara Desert to the European Alps. SDEs are regularly monitored in real-time at the Jungfraujoch meteorological station. Such events are very common in spring (March-June) and autumn (October-November) but rare in summer and winter (Collaud Coen et al., 2004; Papayannis et al., 2008; Conen et al., 2015; Flentje et al., 2015). However, an exceptionally long-lasting (40 h) winter SDE deposited dust particles originating from the Sahara Desert on snow in the Swiss Alps in February 2014. The SD particles and the associated bacteria were covered by fresh snow 3 days after deposition. This rare winter SDE offered the unique opportunity to sample a well-preserved SD-layer within the snowpack. In addition to the winter SDE, a shorter (22 h) spring SDE in May 2014 deposited dust particles just before snowmelt. Together, these deposits allow for the comparison of SDEs occurring in different seasons and originating from different regions of the Sahara Desert.

The aim of this study was to characterize bacterial communities within the SD-layers preserved in snow through a combination of high throughput sequencing and incubations for microbiological metabolic activity using MiSeq Illumina® and Biolog EcoPlate™, respectively. We compared different layers from a snow profile taken at Jungfraujoch in which we could distinguish snow layers containing SD particles from the adjacent clean snow layers (CS-layers). We present the first study to achieve a high resolution sequencing depth and coverage for SDE-associated bacteria in combination with extensive *in situ* and laboratory analyses of physicochemical and geochemical characteristics of the snow and the particles therein.

## Materials and methods

### Site description and sample collection

Sampling was conducted at the Jungfraujoch region (46°33′11″N/8°0′17″E, 3621 m a.s.l.) on a snow field between the high-alpine Jungfraujoch World Meteorogical Station (No. 06730) and the alpine hut Mönchsjochhütte.

A vertical trench was excavated by a snow groomer to a depth of 220 cm below the snow surface on 8 June 2014 at 08:00 CEST. Three snow profiles (A, B, and C) at 100 cm distance to each other were selected for sampling on the shadowy side of the trench. Three replicates for physicochemical and microbiological analyses were taken from the snow profiles by cutting a fresh trench in the snow with a knife sterilized with 70% ethanol. At each profile, a 3.5 × 13 × 13 cm block of snow was cut at seven depths (J0: −25 cm, J1: −80 cm, J2: −120 cm, J3: −145 cm, J4: −150 cm, J5: −155 cm, J6: −170 cm, and J7: −190 cm) and stored in a sterile freezer bag (Supplementary Figure 1C).

Samples were stored in an ice box in the field and transported to the laboratory, where they were stored at 0°C. Snow was melted slowly and subsequently transferred into autoclaved laboratory Schott glass bottles and stored at 0°C until further analyses (total volume between 300 and 480 ml per field sample).

### Physicochemical parameters

Physical parameters such as snow temperature and density were measured *in situ* along the snow profile at 10 and 20 cm intervals, respectively. Temperature was measured with a Testo 925 temperature sensor (Testo AG, Mönchaltdorf, Switzerland). Density was measured with a snow-weighing method (Goodison et al., 1981). Conductivity and pH were measured in homogeneously mixed meltwater at room temperature with a Universal Pocket Meter Multi 350i (WTW, Weilheim, Germany) following the manufacturer's instructions.

### Backward trajectory calculation

Backward trajectories of air masses were calculated using the Vertical Velocity NOAA HYSPLIT Model and GDAS1 meteorological data (Draxler and Rolph, 2003). SDEs were detected by the Paul-Scherrer-Institute (PSI) using the nephelometer aSSA BR TSI method at the Jungfraujoch as described in Collaud Coen et al. (2004). We assessed the intensity of the SDEs by considering the area of the negative albedo scattering.

### Meteorological data

Daily maximum and night minimum air temperature 2 m above the ground was measured at Jungfraujoch (46°32′51″N/7°59′07″E; 3580 m a.s.l.). No snow height data are available from Jungfraujoch. However, snow height was assessed by averaging the data from two meteorological stations at 8.6 km NW and 15 km SE distance to the Jungfraujoch, respectively: Männlichen (46°36′47″N/7°56′27″E; 2343 m a.s.l.) and Eggishorn (46°25′36″N/8°05′34″E; 2927 m a.s.l.). Data were supplied by the Federal Office of Meteorology and Climatology MeteoSwiss (IDAweb).

### Dust particle characterization and quantification

Meltwater (5 ml) containing homogeneously suspended dust particles were filtered through a 0.2 μm GTTP polycarbonate filter with a vacuum pump (Millipore, Billerica, MA, USA). Samples containing high dust concentrations (J0 and J4) were diluted 1:10 to reduce particle density on the filter. The same procedure was applied to three bedrock samples sampled within 200 m of the snow profiles (Rock1: 46°33′9″N/8°0′9″E; Rock2: 46°33′16″N/8°0′27″E, Rock3: 46°33′16″N/8°0′20″E) after being ground to powder.

Dust particles were characterized by scanning electron microscopy (SEM) (Jeol 6390LA) at the Institute of Geochemistry and Petrology (ETH, Zurich). Particle chemical analyses from carbon-coated samples mounted on polycarbonate filters were obtained by energy-dispersive X-ray spectroscopy (SEM-EDX), using a solid state EDS detector combined with the Thermo scientific software NSS3 under high vacuum conditions. The SEM was initially equipped with a W-filament that was later changed to an LAB6 crystal. An acceleration voltage of 15 kV was employed for all analyses with a beam current of 2.5 nA and a probe diameter of 1 μm. The chemical composition of each particle was measured during 15 s live time. Due to the small size of the dust particles, the excitation volume occasionally exceeded the size of the dust particles, which resulted in analytical artifacts that could not be circumvented. All analyses are therefore normalized to a carbon-free basis.

Mineral phases were defined based on a modified plot of Moreno (2003): quartz and low-Al-content silicates (x: 1.0–1.3, y: 0.0–0.2); feldspars (x: 0.8–1.2, y: 0.2–0.5); illite (x: 1.3–1.6, y: 0.6–0.9); kaolinite (x: 1.6–2.5, y: 0.85–1.2); montmorillonite (x: 1.3–1.5, y: 0.4–0.6).

Organic and inorganic particles were quantified using a BD Accuri C6 flow cytometer (BD Biosciences, San Jose, USA). Meltwater (200 μl) was fixed with glutaraldehyde (Sigma-Aldrich, Buchs, Switzerland), stained with 2 μl of Sybr® Green (Life Technologies, Zug, Switzerland) and incubated for 10 min at 37°C.

### Isolation of total DNA

In an attempt to improve the recovery of DNA from spore-forming bacteria and to compensate for low biomass concentrations, two slightly different DNA extraction methods were applied.

Method MOBIO UltraClean: 100 ml of homogeneously mixed meltwater was filtered through a 0.2 μm sterile nylon membrane with a vacuum filtration system (model 87006-076, VWR, PA, USA) (Supplementary Figure 1D). Total DNA was isolated using the MOBIO UltraClean™ DNA Isolation Kit (Mobio Laboratories, CA, USA) according to the manufacturer's instructions with the following modifications: filters were washed over night in a tube (Falcon 15) with 60 μl of solution S1, 600 μl of solution IRS and 400 μl of bead solution. To completely detach the particles from the filters, the tubes were horizontally centrifuged for 30 min at maximum speed. Thereafter, the tubes were sonicated for 10 s in a water bath at room temperature and immediately placed on ice for 20 s. Sonication was repeated twice. Finally, liquid was transferred into the bead-containing tubes, incubated for 10 min at 65°C and processed following the manufacturer's instructions.

Method MOBIO PowerWater: The remaining volume (variable in each sample between 20 and 280 ml) of homogeneously mixed meltwater was filtered through a 0.2 μm sterile nylon membrane with a vacuum filtration system (model 87006-076, VWR). Due to plugging, two filters were used for samples containing SD particles. Filters were cut in two halves. One half of each filter was used for extraction using the MOBIO PowerWater™ DNA Isolation Kit (Mobio Laboratories) according to the manufacturer's instructions with the following modifications: PowerWater® Bead Tubes were heated at 65°C for 10 min as indicated by the alternate lysis method. Thereafter, the tubes were shaken horizontally for 1 h at maximum speed. DNA was eluted in 50 μl PW6 and stored at −20°C.

The DNA of all three extractions (1 extraction with method MOBIO UltraClean and 2 extractions with method MOBIO PowerWater) was pooled prior to further processing.

Extracted DNA was quantified with the Qubit® Fluorometer dsDNA HS Assay Kit and normalized to the total volume of the filtered meltwater.

### Low cycle amplicon PCR

Multiplex MiSeq Illumina® sequencing was performed with low-cycle amplification, Nextera indexing per the instructions of the Genetic Diversity Center (GDC; ETH Zurich, Switzerland).

For PCR amplification, the universal forward primer Bakt_341F (Accession Number pB-03844) 5′-CCTACGGGNGGCWGCAG-3′ and universal reversed primer Bakt_805R (pB-03845): 5′-GACTACHVGGGTATCTAATCC-3′ (Herlemann et al., 2011) were modified for subsequent MiSeq Illumina® sequencing of the 16S rRNA gene hypervariable region V3–V4 by adding the overhang (italic) and an insertion of zero to three nucleotides between the overhang and the primer sequence (bold; Supplementary Table 1).

Each replicate sample was amplified in four separate PCR reactions with the four different primer pairs (fs0, fs1, fs2, and fs3). 20 μl of PCR reaction mixture was composed of 1 × KAPA Sybr® Fast Universal qPCR Mix (KAPA Biosystems, MA, USA), 400 nM of each primer (Microsynth, Balgach, Switzerland), DEPC-water (Roth, Karlsruhe, Germany) and 2 μl of DNA template. Low cycle amplicon PCR was performed using a Labcycler (SensoQuest, Göttingen, Germany) thermal cycler with the following program: denaturation at 95°C for 5 min followed by 10 cycles of 95°C for 30 s, annealing at 55°C for 30 s and extension at 72°C for 30 s. A final extension step was run at 72°C for 5 min. The PCR products of each sample with the four different primer pairs (fs0, fs1, fs2, and fs3) were pooled and subsequently purified with 0.8 × Agencourt AMPure XP Kit (Beckman Coulter, CA, USA) following the manufacturer's instructions. Purified PCR products were resuspended in 30 μl of DEPC-water.

Nextera XT Index PCR was performed in 50 μl of PCR mixture composed of 1 × KAPA Sybr® Fast Universal qPCR Mix (KAPA Biosystems), 5 μl of Nextera forward (N7XX) and reverse (S5XX) indices and 15 μl of previously purified PCR product. Index PCR was performed as described above, but with 8 cycles instead of 10. PCR products were purified as described above with 1 × Agencourt AMPure XP Kit (Beckman Coulter).

Purity of the index PCR products was checked with the Bioanalyzer High Sensitivity Chip (Agilent Technologies, Santa Clara, USA). Concentration of the index PCR products was determined with the Qubit® Fluorometer dsDNA HS Assay Kit and by qPCR with the KAPA Library Quantification Kit for MiSeq Illumina® (KAPA Biosystems) following manufacturer's instructions. Samples were pooled by adding 0.75 nM of each sample to the library, which was subsequently concentrated to 4 nM with the 1 × Agencourt AMPure XP Kit (Beckman Coulter).

### High throughput amplicon sequencing

Paired-end (2 × 300 nt) high throughput sequencing of PCR amplicons of the 16S rRNA gene was performed in a single multiplexed run on an Illumina MiSeq (Illumina® Inc., San Diego, USA; software v2.4.1.3) at the GDC. A total of 4,095,381 sequences were obtained from 23 samples. The sequences of the field replicates A–C were merged resulting in 8 samples: J0: 462,608 reads; J1: 543,977; J2: 107,085; J3: 88,383; J4: 961,545; J5: 198,260; J6: 1,108,814; J7: 624,709. All results based on HTS reported in this study are based on these 8 samples. 16S rRNA gene data processing was performed following the pipeline designed at the GDC (Supplementary Table 2). Sequences were clustered at 97% sequence similarity's level and defined as Operational taxonomic unit (OTU) with Uparse in Uchime (Edgar, 2013). Only OTUs detected more than 3 times were considered for community analysis, reducing the complexity from 637 to 539 OTUs. The sequence reads obtained in this study were deposited in the European Nucleotide Archive (ENA) with the project number PRJEB9478 (www.ebi.ac.uk/ena/data/view/PRJEB9478).

### Real-time quantitative PCR

Quantification of 16S rRNA gene copies was performed by real-time quantitative PCR (qPCR) on an ABI 7500 system (Applied Biosystems) at the GDC. 20 μl of the qPCR reaction mixture contained 1 × KAPA Sybr® Fast Universal qPCR Mix (KAPA Biosystems), 400 nM of each primer, forward B341F_fs0 and reverse B805R_fs0 (Microsynth, Balgach, Switzerland), DEPC-water (Roth, Karlsruhe, Germany) and 1 μl (0.2–43 ng/μl) of DNA template per reaction. Triplicates of no-template controls, containing DEPC-treated water, were included. Thermal cycles were performed using the following program: denaturation at 95°C for 10 min followed by 40 cycles of 95°C for 20 s, annealing at 55°C for 30 s, and extension and acquisition at 72°C for 1 min. A final extension step was run at 72°C for 5 min.

As bacterial standard, dilution series of PCR products derived from DNA extracted from the control strain *Methylococcus capsulatus* (strain Bath; courtesy of Prof. Svenning, University of Tromsø, Norway) in a range between 2.12 e^1^ and 2.12 e^8^ was used. The 16S rRNA gene copy number was calculated from the standard curves, assuming that the average molecular mass of a double stranded DNA molecule is 613 g mol^−1^. The 16S rRNA gene copy number was within the range of the standard curve for all samples.

### Multivariate and statistical numerical analyses

Ordination analysis by non-metric multidimensional scaling analysis (NMDS) was performed by applying Bray-Curtis dissimilarity algorithm to the lowest total count (88,383 reads of J3) normalized community composition matrix with the 7381 OTUs (Legendre and Anderson, 1999; Anderson et al., 2011). To show differences in bacterial community structure between all field samples, the NMDS was performed using the package “phyloseq” (version 1.8.2) (McMurdie and Holmes, 2013) in the R program (version 3.1.2) (R Core Team, 2015). Each OTU is represented by a dot and to facilitate visualization, the NMDS was split based on the taxonomic affiliation of the OTUs at phylum level. Adjusted statistical significance was calculated using one-way analysis of variance (ANOVA) and a false discovery rate correction (Benjamini and Hochberg, 1995). The values of all field replicates for particle counts, conductivity, 16S rRNA gene copies, DNA were log10-transformed (*n* = 2 for J0; *n* = 3 for J1–J7).

The indices of biological diversity richness, E evenness, J′ evenness, Shannon, Simpson, Inverse Simpson and Berger were calculated using the package “vegan” 2.3-0 (Oksanen et al., 2015) and “BiodiversityR” 2.5-3 (Kindt and Coe, 2005). No error bars are shown because diversity indices were calculated after the merging of the sequences of each field replicate. Standard deviations among field replicate might not reflect biological variability but rather artificial variability of the amplicon HTS.

### Phylogenetic tree

A phylogenetic tree containing 539 OTUs aligned with PyNAST (Caporaso et al., 2010) was built using Maximum Likelihood method and the software FastTree 2.1 (Price et al., 2010). The tree was visualized with ITOL (Letunic and Bork, 2011).

### Ecophysiology

Biolog incubations were performed by inoculating 200 μl of homogeneously mixed meltwater from each snow layer field replicate (triplicates for each layer) in a Biolog EcoPlate™ (BIOLOG, CA, USA). Incubation was performed at 4°C for 36 days. Color development was assessed twice a day by measuring the optical density (OD) of the samples at 595 nm with a Biotek plate reader (Bio-TeK, VT, USA). The time point of maximum Average Well Color Development (AWCD) was observed after 739 h (30.8 days) of incubation. AWCD for each time point was calculated according to the formula described in Lazzaro et al. (2015).

Samples J0 and J4 were sterilized by CHCl_3_ fumigation to test whether the increasing OD_595_ measured during the Biolog incubation was influenced by dust particles, by active exoenzymes or by metabolically active bacterial cells. We fumigated 7 ml of meltwater in glass beakers as described in Blankinship et al. (2014) for 1 day. Biolog incubation was performed as described above.

## Results

### Source and deposition of Sahara dust particles

**Figure 1:**
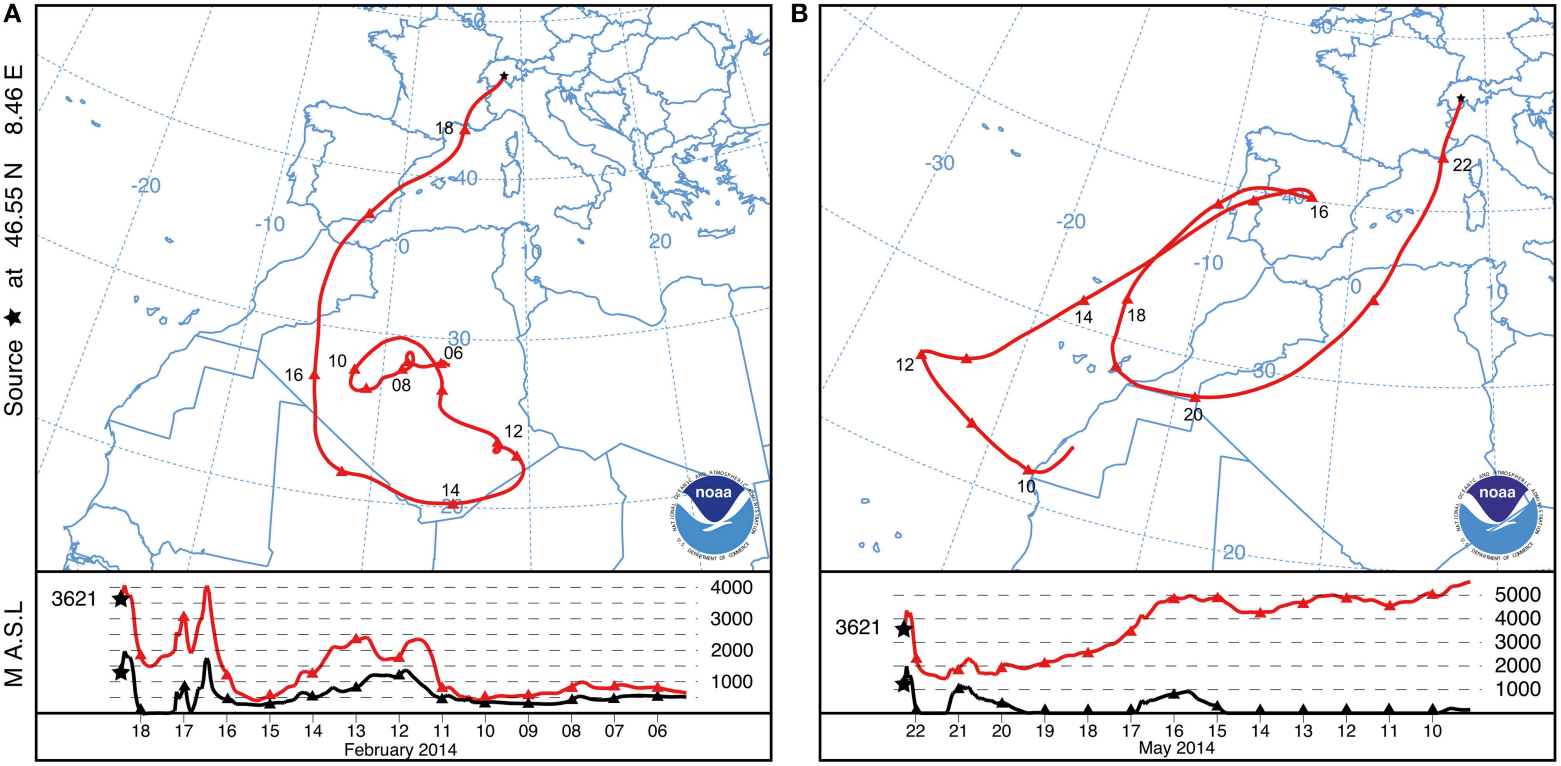
**NOAA HYSPLIT backward trajectory of air masses that reached the sampling site at Jungfraujoch (3621 m a.s.l.; black star)**. Only one representative trajectory is shown out of 40 and 22 trajectories for each hour of the February and May SDE length, respectively. The lower panel shows the vertical trajectory of the air masses (red line) with respect to the relief (black line). **(A)** One selected characteristic trajectory of the SDE in February 2014. **(B)** One selected characteristic trajectory of the SDE in May 2014.

We report here a strong SDE detected by the method described in Collaud Coen et al. (2004) at the Jungfraujoch in February 2014. The winter SDE lasted 40 h, starting on 18 February 2014 at 05:03:50 (CET) and ending on 19 February 2014 at 21:04:16 (CET). Backward trajectories indicate that south-central Algeria is the likely source region of the aerosols deposited during the SDE. Neighboring countries including Niger, Mali and Morocco (Figure 1A) may have also contributed dust particles. Uplifted SD particles were transported to the Alps without any further contact with the ground until deposition on the snow at the Jungfraujoch (Figure 1A; lower panel). Meteorological records indicate that the SD particles were covered by fresh snow 3 days after being deposited at freezing temperatures between −17.1°C and −6°C (Supplementary Figure 2B).

Between the February SDE and the sample collection date (8 June 2014), an additional SDE was detected at the Jungfraujoch starting on 21 May 2014 at 20:44:25 (CET) and lasted 22.4 h until 22 May 2014 at 19:06:38 (CET). Backward trajectories indicate that aerosols of the spring SDE likely originated from the northwestern part of the Algerian desert (Figure 1B). The SD particles of the spring SDE were deposited just before snowmelt at temperatures between −4.4°C and −0.9°C (Supplementary Figure 2A). Based on the negative exponent of the single scattering albedo, the winter SDE was about 6 times as intense as the spring SDE.

### Snow profile and physicochemical parameters

**Figure 2:**
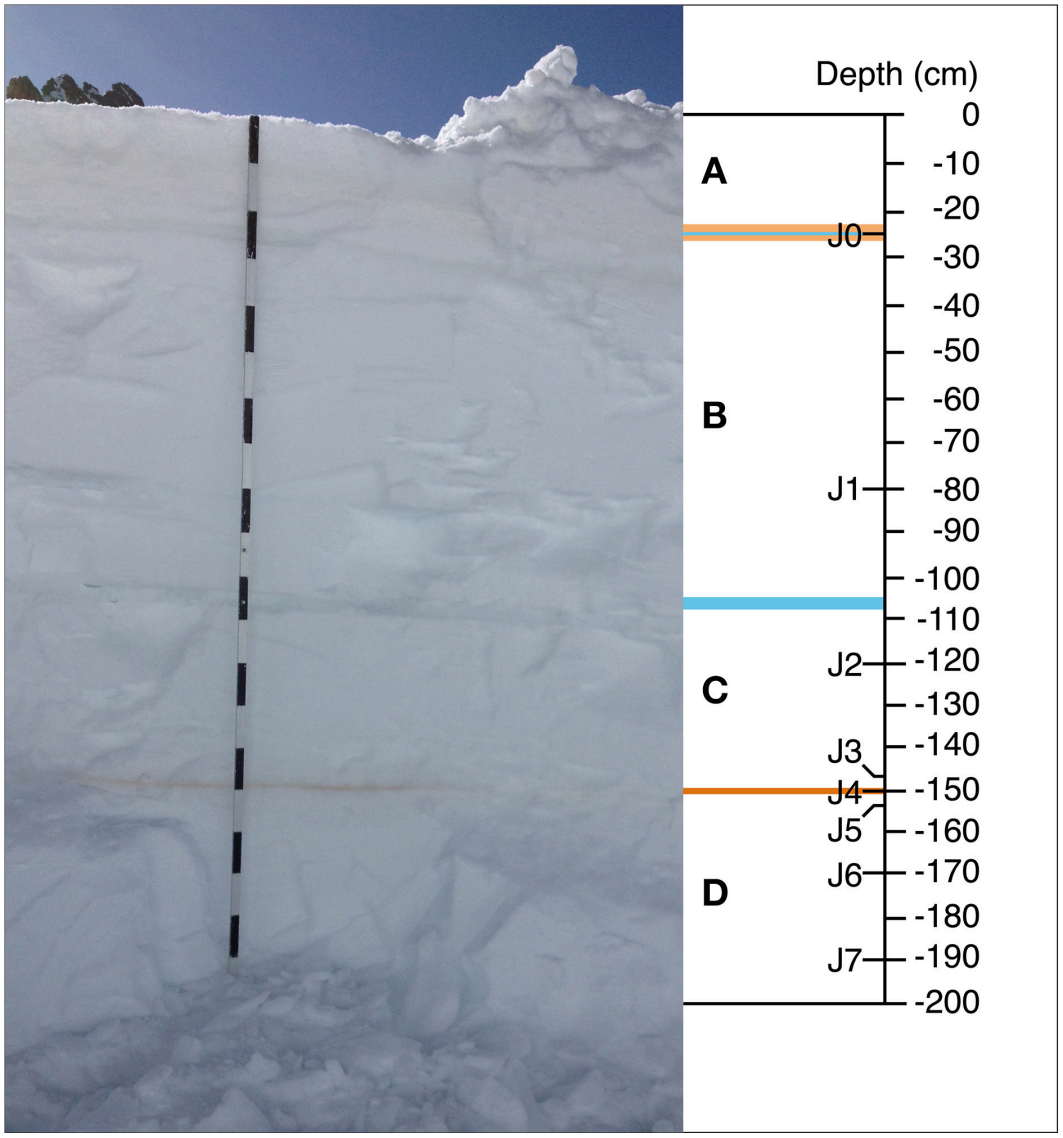
**Section of one vertical snow profile sampled at Jungfraujoch**. **(A–D)** the observed four different snow layers. **(A)** 24 cm thick and limited at the top by the snow surface and at the bottom by a 0.3 cm thick ice lens (blue) located within a faint, ochre-colored 3.5-cm thick layer that corresponded to sample J0. **(B)** 78.5 cm thick, ranging in depth from −26.5 to −105 cm, delimited at the bottom by a 2.5-cm thick ice lens (blue) associated with surface exposure during a period with no snowfall in April. **(C)** 42.5 cm thick; ranging in depth from −107.5 to −150 cm and delimited at the bottom by a distinct, ochre-colored layer 0.6 cm thick. That layer corresponded to sample J4. **(D)** 50 cm thick, ranging in depth from -150 to -200 cm. The sample J0 sample was collected in field duplicates, J1–J7 in field triplicates.

The 200-cm deep snow profile displayed four distinct snow layers (Figure 2). The distinct ochre color of the two thin bands designated as J0 and J4 at −25 cm and −150 cm, respectively, allowed us to ascribe these deposits to potential SDEs. For simplicity, we hereafter refer to J0 and J4 as SD-layers and to all other samples (J1, J2, J3, J5, J6, and J7) as CS-layers. SD-layer J4 was unaffected by freeze-thaw cycles but slightly altered by snow compaction (Supplementary Figure 1B). In contrast, J0 was affected by freeze-thaw cycles in the days immediately prior to sampling, as air temperature rose above freezing during the day (Supplementary Figure 2B). On the day of sampling, the freezing point was measured at −25 cm, within SD-layer J0 (Supplementary Figure 1A).

**Figure 3:**
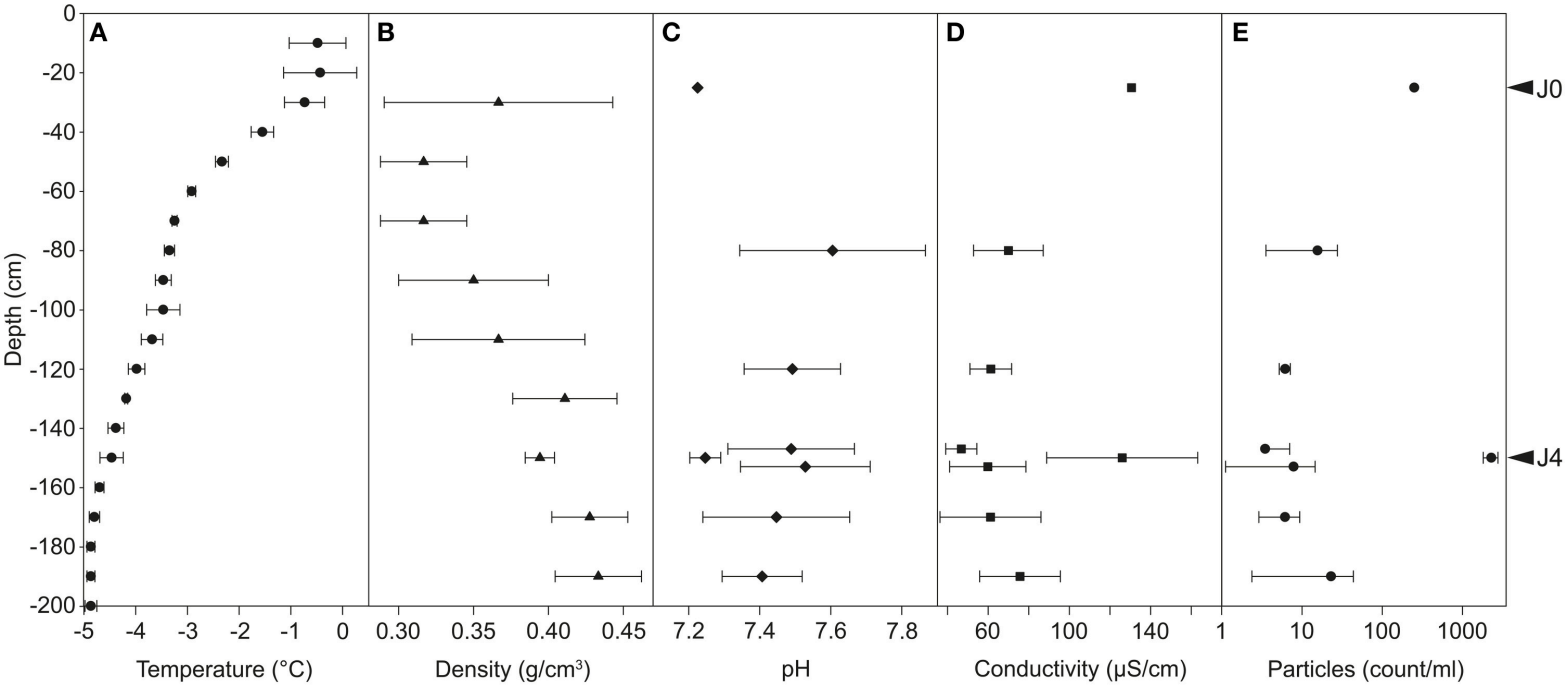
**Physicochemical properties of the snow profiles at Jungfraujoch**. Temperature **(A)** and density **(B)** were measured in the field at 10- and 20-cm intervals, respectively. pH **(C)** and conductivity **(D)** were measured in meltwater in laboratory conditions. Particles suspended in meltwater were counted by flow cytometry (**E**; common Log scale). Error bars represent only positive values of standard deviation.

Temperatures in the snow profile varied from −0.4 to −4.9°C and decreased with increasing depth (Figure 3A). The temperature in layer A was around −0.5°C, between −0.7 and −3.5°C in layer B, between −3.5 and −4.5°C in layer C and below −4.5°C in layer D. Snow density increased with increasing depth from 0.32 to 0.43 g cm^−3^; two deviations from this trend were found at −30 cm and −150 cm (Figure 3B).

The pH in the SD-layers was around 7.2, and significantly lower (*P* < 0.01) than that of CS-layers (pH 7.4-7.6; Figure 3C). A significant difference was also observed for conductivity (*P* < 0.001; Figure 3D): SD-layers had values between 126 and 130 μS cm^−1^, whereas the conductivity of the CS-layers ranged between 47 and 76 μS cm^−1^.

The dust particle concentration observed in J4 (2300 particles ml^−1^ snow) was two orders of magnitude greater than that of the CS-layers (3–22 particles ml^−1^ snow), and 9.1 times greater than that of J0 (250 particles ml^−1^ snow) (Figure 3E). The SD-layers were significantly enriched in dust particles compared to the CS-layers (*P* < 0.00001).

### Geochemical and mineralogical analyses

**Figure 4:**
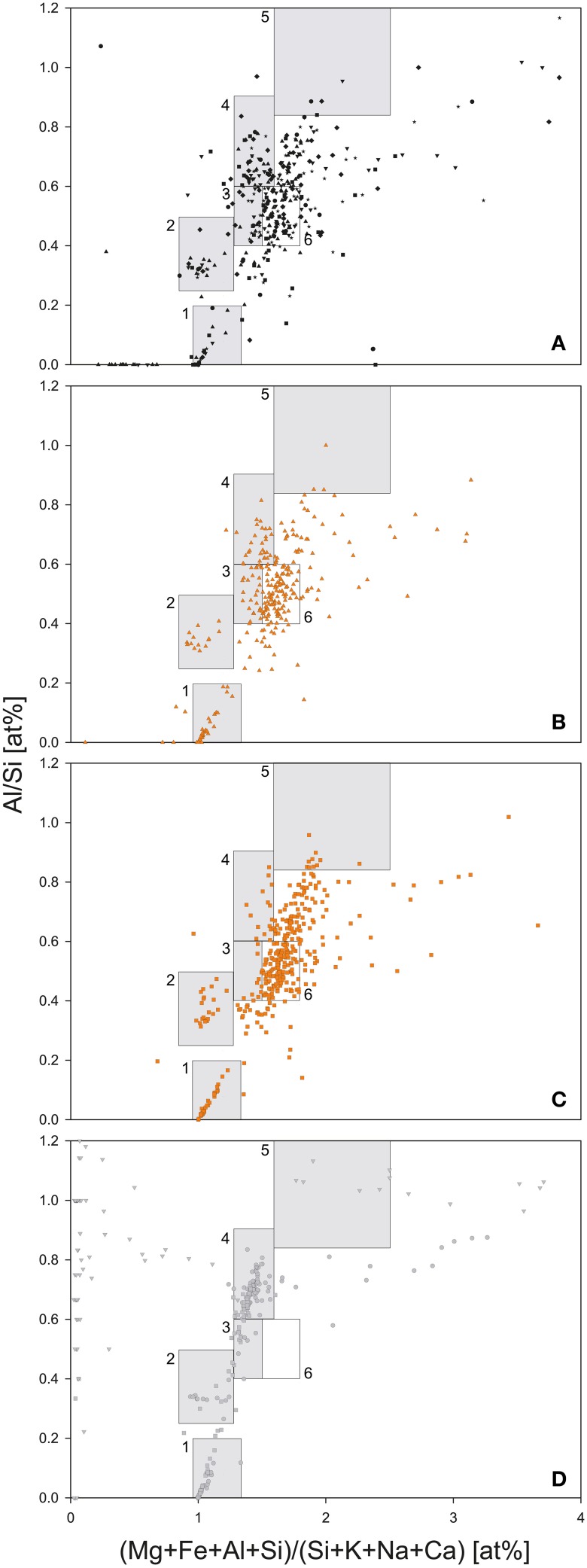
**Geochemical analysis of dust particles and bedrock samples by SEM-EDX**. **(A)** CS-layers (J1, J2, J3, J5, J6, and J7). **(B)** SD-layer J0. **(C)** SD-layer J4. **(D)** Bedrock samples Rock1 (circles), Rock2 (squares), and Rock3 (triangles). Boxes represent potential mineral phases: (1) quartz; (2) feldspar; (3) montmorillonite; (4) muscovite and illite; (5) kaolinite; (6) Range of particles enriched in SD-layers J0 and J4 as compared to clean snow and bedrock samples.

**Figure 5:**
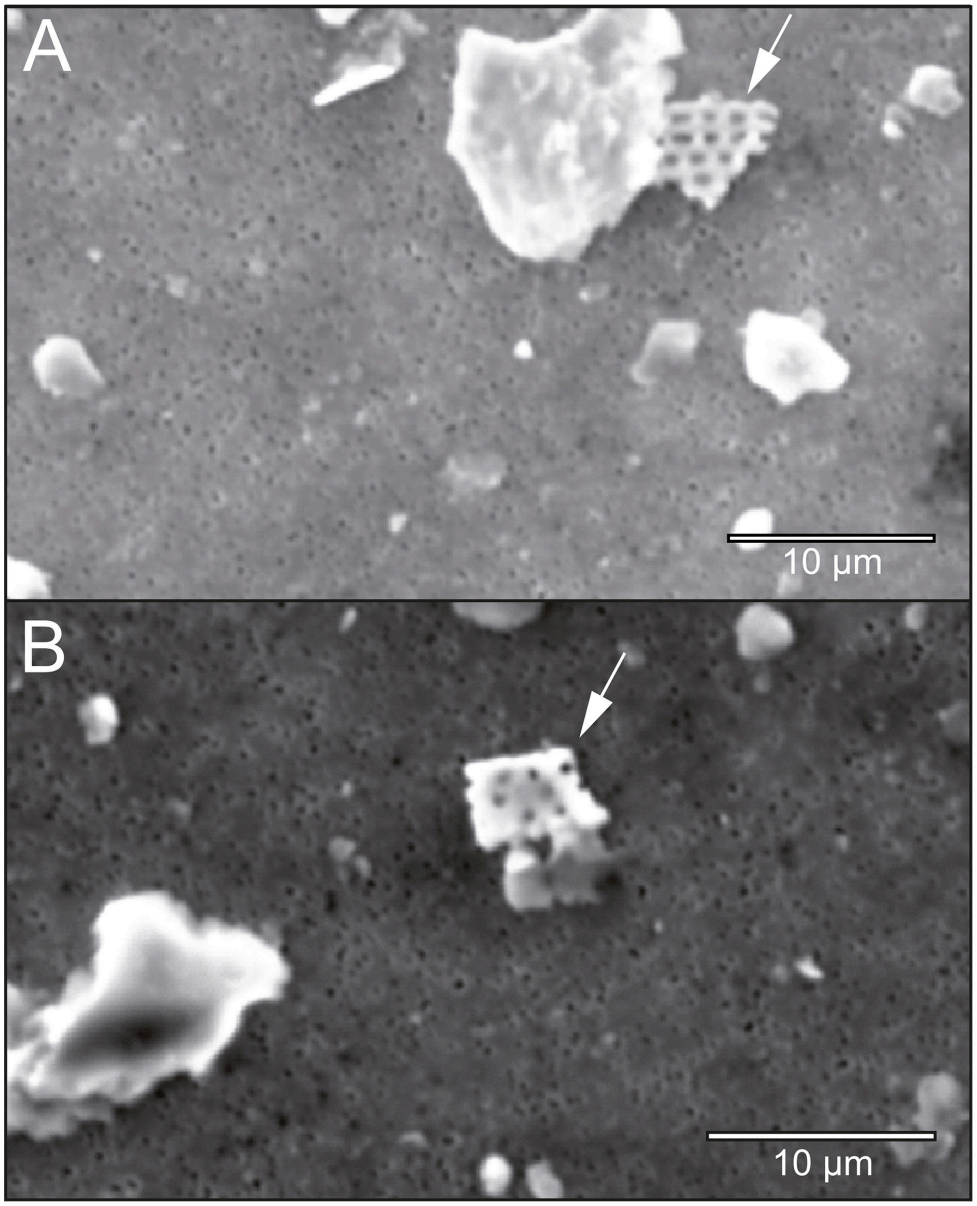
**Diatom fragments (arrow) detected on two different field replicates of J4 under the SEM**. A diatom fragment in sample J4A **(A)** and J4B **(B)**.

Two out of three bedrock samples collected at Jungfraujoch were gneisses, Rock1 and Rock2, whereas Rock3 was a limestone (Supplementary Table 3), which is evidence of the geological heterogeneity of the Jungfraujoch region (Labhart, 2001). The felsic mineral particles in the gneisses exhibited a low variety of mixed-phases and could therefore be easily assigned to a typical mineral phase cluster, whereas the mineral particles of the limestone plotted outside the defined clusters of felsic minerals (Figure 4D). In contrast, the deposited SD particles contained a larger variety of mixed-phase minerals (Figures 4B,C). In comparison to the CS-layers (Figure 4A), SD-layers had higher particle concentrations (Figure 3E). Most of these particles plotted between 1.5 and 1.8 on the x-axis and 0.4–0.6 on the y-axis and could not be assigned to any mineral phase (box nr. 6 in Figures 4B,C). These particles represented approximately 34% of all particles in the SD-layers but only 16.6 ± 5.6% in the CS-layers and were completely absent in the bedrocks. J0 contained more illite particles than J4 (8.7 vs. 4.4%) but less kaolinite particles (0.7 vs. 2.3%). The I/K ratio was therefore higher in J0 than in J4 (6.75 vs. 1.21). The (Ca+Mg)/Fe [wt.%] ratio was similar in both SD-layers (J0: 0.41; J4: 0.39). Finally, diatoms were detected exclusively in J4 (Figure 5).

### Biomass and diversity indices

**Figure 6:**
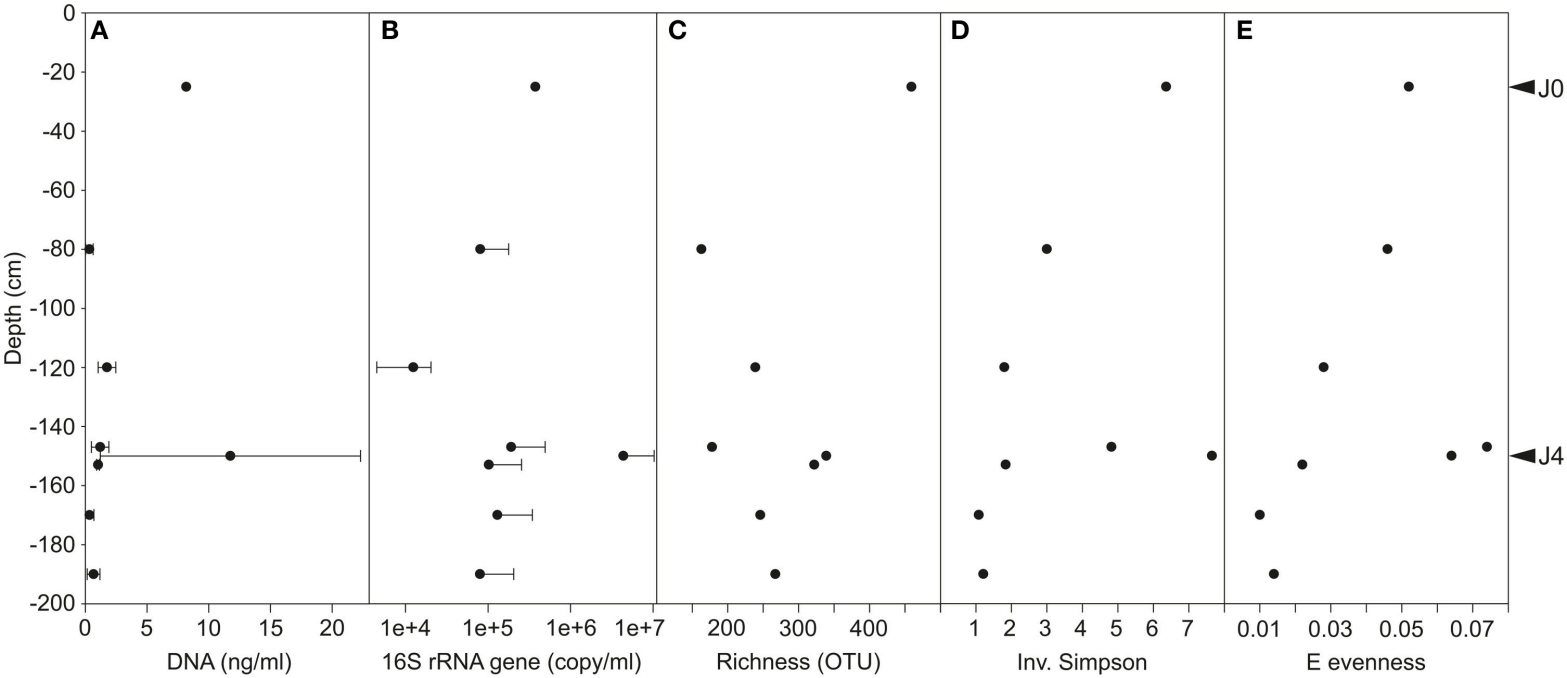
**(A)** DNA concentration. Variability was high in J4 where DNA concentration ranged between 4.5 and 23.8 ng ml^−1^ meltwater. **(B)** 16S rRNA gene copies per ml of meltwater (common Log Scale). Error bars represent only positive values of standard deviation. Diversity indices of Richness **(C)**, Inverse Simpson **(D)**, and E evenness **(E)** on bacterial communities of merged field replicates, therefore no standard deviations are shown.

The SD-layers contained similar average quantities of DNA per volume of meltwater (J0: 8.2 ng ml^−1^ and J4: 11.8 ± 10.6 ng ml^−1^), and significantly more DNA (*P* < 0.001) than the CS-layers (0–1 ng ml^−1^; Figure 6A). Although DNA was more abundant in J4 than in J0 by a factor of 1.4, the number of 16S rRNA gene copies per ml meltwater was 10.9 times higher in J4 as compared to J0. However, despite that difference, the SD-layers contained a significantly (*P* < 0.001) higher amount of 16S rRNA gene copies (J0: 7.9 × 10^5^ copies ml^−1^ meltwater; J4: 8.7 × 10^6^ copy ml^−1^ meltwater) than the CS-layers (1.0–4.2 × 10^5^ copies ml^−1^ meltwater; Figure 6B). DNA concentration (*R*^2^= 0.62, *P* < 0.00001) and 16S rRNA gene (*R*^2^ = 0.6, *P* < 0.0001) copies were both positively correlated with particle concentrations.

The species richness was significantly higher in the SD-layers (J0: 459 OTUs; J4: 339 OTUs; *P* < 0.05) than in the CS-layers (163–322 OTUs; Figure 6C), but no significant correlation between richness and depth was observed. Despite the greater richness of the SD-layers, a higher concentration of bacterial cells was not detected by flow cytometry (data not shown). Inverse Simpson (*P* < 0.01) and Shannon (*P* < 0.05) values were significantly higher in SD-layers (inverse Simpson: J0: 6.4, J4: 7.7; Shannon: J0: 2.5, J4: 2.4) than in the CS-layers (inverse Simpson: 1.1–4.8; Shannon: 0.2–1.9) (Figure 6D, Supplementary Figure 3B). However, no significant difference between in Simpson and Berger diversity could be identified between SD- and CS-layers (Supplementary Figures 3A,C). E and J′ evenness decreased with depth except in J3 and J4 (Figures 3D, 6E) and was similar in the SD-layers (J0: 0.026, J4: 0.032). Despite decreasing diversity and evenness values, no significant Spearman's correlations between these indices and depth could be observed, which was mainly due to the anomalous values in J4.

### Bacterial community analysis

**Figure 7:**
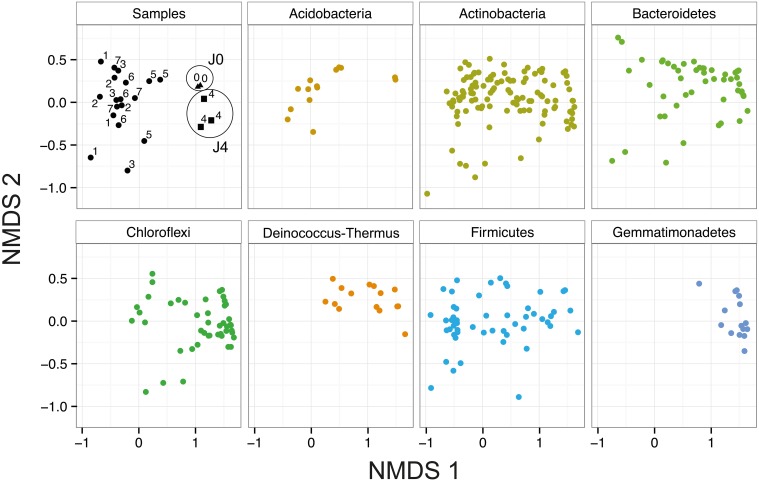
**Bacterial community composition in snow at Jungfraujoch**. NMDS analysis of the bacterial communities based on Bray–Curtis dissimilarity of all 23 field sample replicates (Two factor stress value = 0.14). Samples J0 (triangles) and J4 (squares) are highlighted with circles.

In the NMDS, J0, and J4 clustered together and were distinct from the other samples (Figure 7). This difference is mostly explained by the axis NMDS1, which results from the presence of a few phyla in the SD-layers, including *Gemmatimonadetes, Deinococcus-Thermus* and certain *Bacteroidetes* and *Chloroflexi*. The phyla with the highest number of phylotypes were the *Proteobacteria, Actinobacteria* and *Firmicutes;* these phyla were equally distributed in all layers. The less well-represented phyla *Acidobacteria* and *Cyanobacteria* were more abundant in the CS-layers than in the SD-layers. The phyla *Elusimicrobia, FBP, OD1, Planctomycetes* and *WPS-2* were each represented by a maximum of four OTUs.

### Bacterial composition

*Proteobacteria* was the most abundant phylum in both SD- and CS-layers (J0: 79.7%; J4: 69.8%; CS-layers: 73.9%; Supplementary Figure 4; Supplementary Table 4). The SD-layers differed from the CS-layers in the composition of *Proteobacteria* at the class level and were dominated by *Betaproteobacteria* (J0: 76.2%; J4: 36.3%; CS-layers: 23.7%). In fact, SD-layers contained a higher abundance of the family *Comamonadaceae* than the CS-layers (J0: 40.3%; J4: 11.6%; CS-layers: < 1%), most of which were related to the genera *Variovorax, Polaromonas* and *Delftia*. The second most abundant *Betaproteobacterium* was *Janthinobacterium*, a member of the family *Oxalobaceraceae*, which was highly abundant in all layers (J0: 15.9%; J4: 24.5%; CS-layers: 18.0%). *Gammaproteobacteria* related to the genus *Pseudomonas* were particularly abundant in the CS-layers and J4 (J0: < 1%; J4: 33%; CS-layers: 49.2%) *Alphaproteobacteria* had low abundances in all samples (J0: 3.2%; J4: < 1%; CS-layers: 1.1%) and was mostly represented by *Sphingomonas*.

*Bacteroidetes* were highly abundant in the SD-layers but negligible in the CS-layers (J0: 6.5%; J4: 29.2%; CS-layers: < 1%). Both SD-layers shared a high abundance of *Hymenobacter* (J0: 6.4%; J4: 9.3%; CS-layers: < 1%), whereas *Flavobacterium* was highly abundant only in J4 (J0: < 1%; J4: 20.0%; CS-layers: < 1%).

Sporulating bacteria such as *Actinobacteria* and *Firmicutes* were present in low abundances. *Actinobacteria* was detected in abundance only in J0 (J0: 10.4%; J4: < 1%; CS-layers: 3.4%) and were mostly represented by *Salinibacterium* and *Micrococcaceae*, two members of the family *Microbacteriaceae*. *Firmicutes*, mostly represented by *Bacilli Staphylococcus*, was detected only in the CS-layers (J0: < 1%; J4: < 1%; CS-layers 2.1%). *Gemmatimonadetes* showed low abundance in the SD-layers but were almost completely absent in the CS-layers (J0: 0.6%, J4: 0.2%; CS: < 0.01%). *Deinococcus-Thermus* was detected at a higher abundance in the SD layers than in other layers (J0: 0.2%, J4: 0.01%; CS: < 0.01%).

### Occurrence of unique OTUs

**Figure 8:**
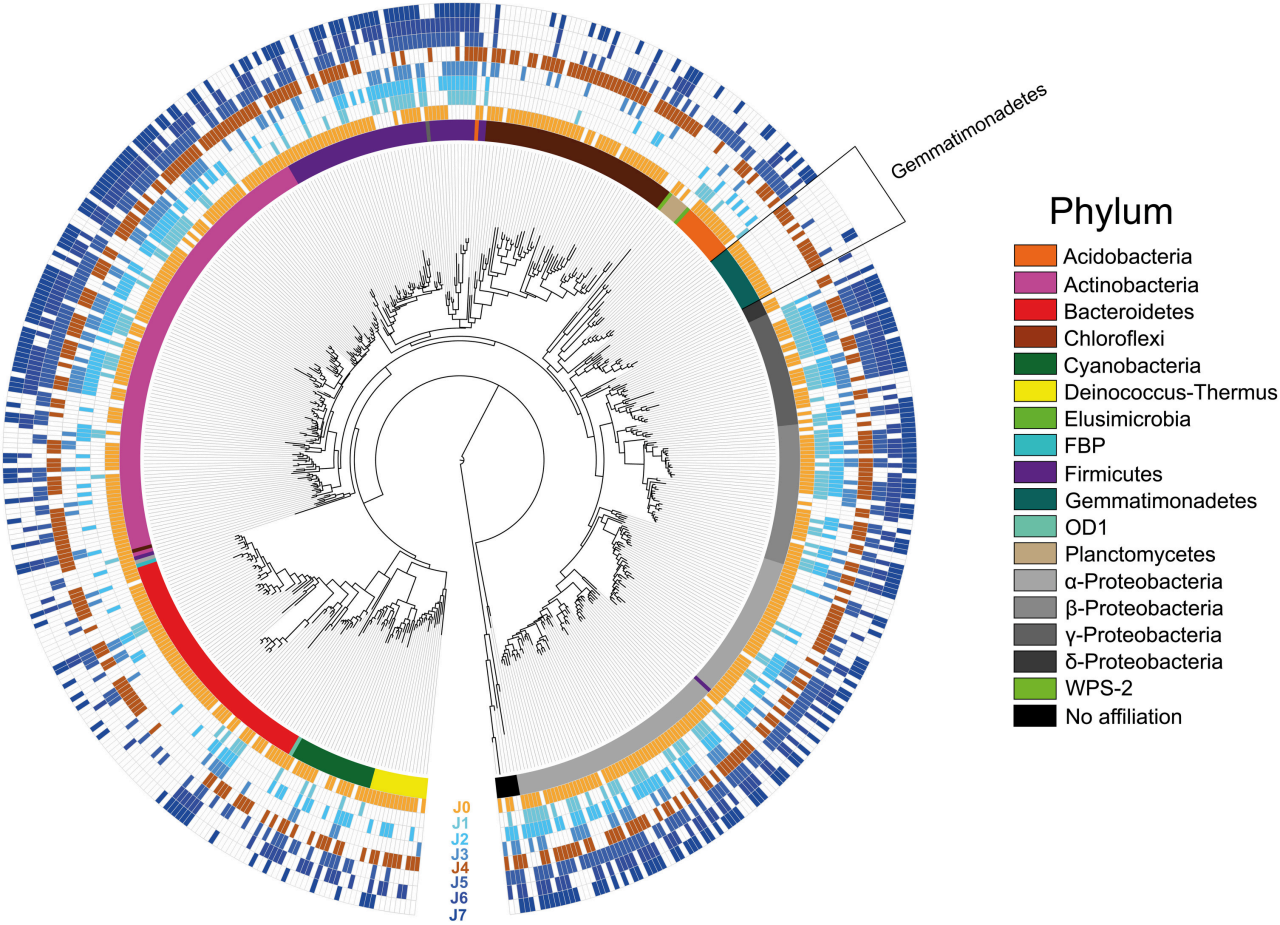
**Most Likelihood phylogenetic tree with presence/absence of all OTUs**. Empty fields portray absence, whereas colored fields portray presence of an OTU. The snow samples collected at different depths are presented with increasing dark blue color. The SD-layers are presented in light brown (J0) and dark brown (J4). Colors of the inner ring correspond to the phyla in the legend.

Considering the overall species richness of OTUs detected in the samples (total 539 OTUs), the most represented phyla were the *Proteobacteria*, followed by *Actinobacteria, Bacteroidetes, Firmicutes, Chloroflexi, Cyanobacteria, Gemmatimonadetes, Acidobacteria, Deinococcus-Thermus, Planctomycetes, Elusimicrobia, FBP, OD1, WPS-1*, and six undetermined phyla (Figure 8; Supplementary Table 5).

**Figure 9:**
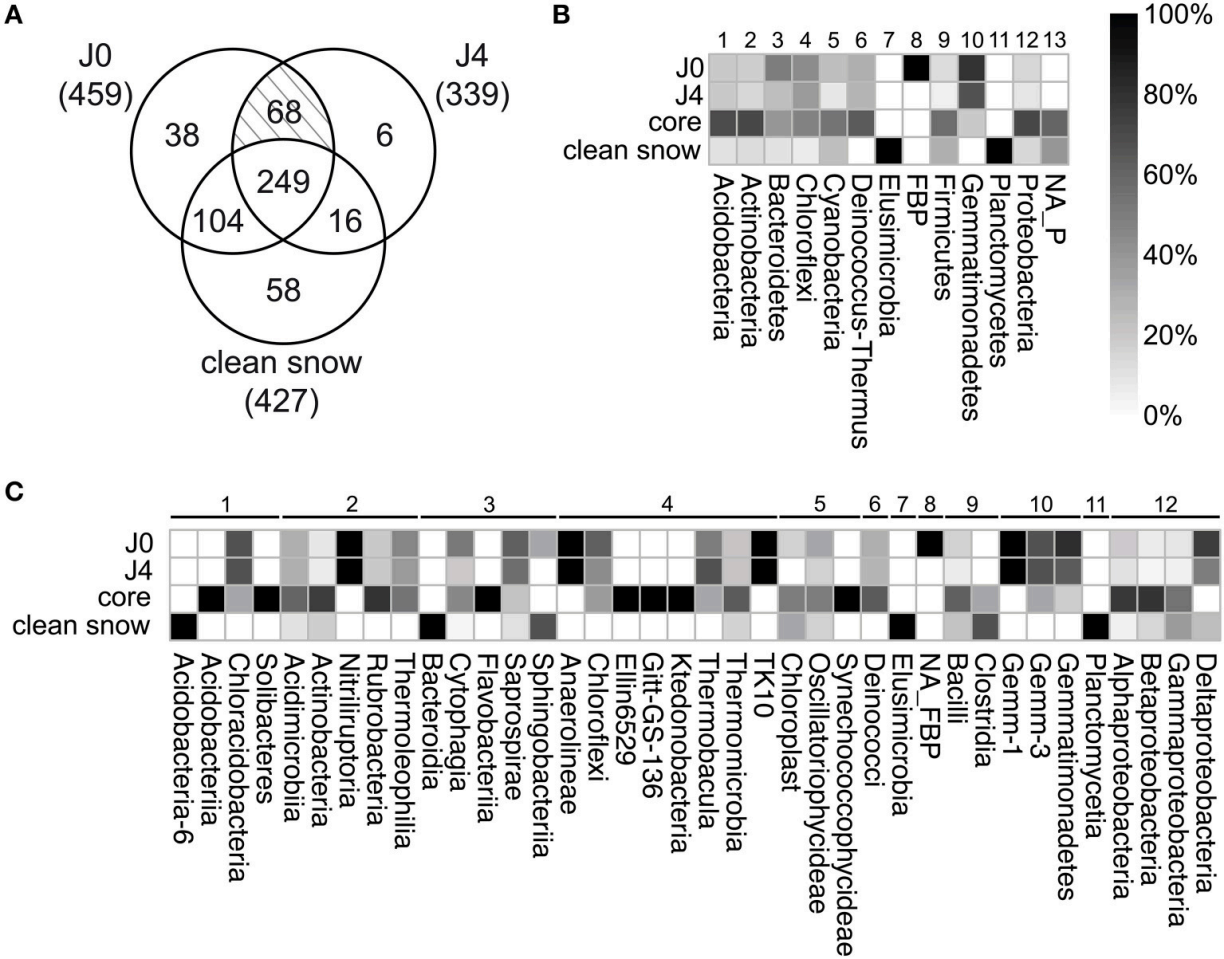
**(A)** Venn diagram visualizing sections with numbers of unique and shared OTUs between SD-layers (J0, J4) and the CS-layers (J1, J2, J3, J5, J6, J7). In brackets, total OTUs for each section. Of a total of 539 OTUs, 249 composed the core community. 38 OTUs were unique for J0, 6 for J4 and 58 for the CS-layers. The shaded section represents shared OTUs between J0 and J4 (68). Relative distribution of unique OTUs in the different samples at phylum **(B)** and class level **(C)**. For core and CS-layers, a total of 249 and 58 OTUs were considered, respectively. For J0 and J4, the 68 shared OTUs were considered unique to both and included in the analysis, resulting in 106 OTUs for J0 (38+68) and 74 OTUs for J4 (6+68). Assigned phyla are numbered 1–12; unassigned phyla are labeled NA_P.

To identify the SDE-specific bacteria, we built a three-factor Venn diagram containing the OTUs of J0, J4, and the CS-layers (Figure 9A). 112 OTUs were specific to either J0 or J4 or were common to both. These OTUs represented 20.8% of all detected OTUs and 23.0% of the 481 J0 and J4 OTUs. Of the 112 OTUs, 26 were *Bacteroidetes* (40.6% of all *Bacteroidetes* OTUs), 23 were *Chloroflexi* (43.4%), 20 *Proteobacteria* (11.9%), 16 *Actinobacteria* (13.7%), 12 *Gemmatimonadetes* (75%), 5 *Firmicutes* (9.3%), 4 *Deinococcus-Thermus* (26.7%), 3 *Cyanobacteria* (13.6%), 2 *Acidobacteria* (13.3%), and 1 *FBP* (100%) (Figure 9B, Supplementary Table 5). At lower taxonomical levels, it is worth noting the overrepresentation of certain classes observed only in the SD-layers, such as the members of the phylum *Gemmatimonadetes: Gemm-1* (100%), *Gemm-3* (66.7%) and *Gemmatimonadetes* (75%), the deltaproteobacterium *Myxococcales* (*75%*), *Deinococci* (26,7%), the acidobacterium *Chloracidobacteria* (40%), the actinobacterium *Nitriliruptoria* (100%) and *Thermoleophilia* (40%), the bacteroidetes *Cytophagia* (42.2%) *and Saprospirae* (66.7%), *Chloroflexi* (58.8%), *Thermobacula* (66.7%), *TK10* (100%), and an unknown class of *FBP* (100%) (Figure 9C).

### Ecophysiology

**Figure 10:**
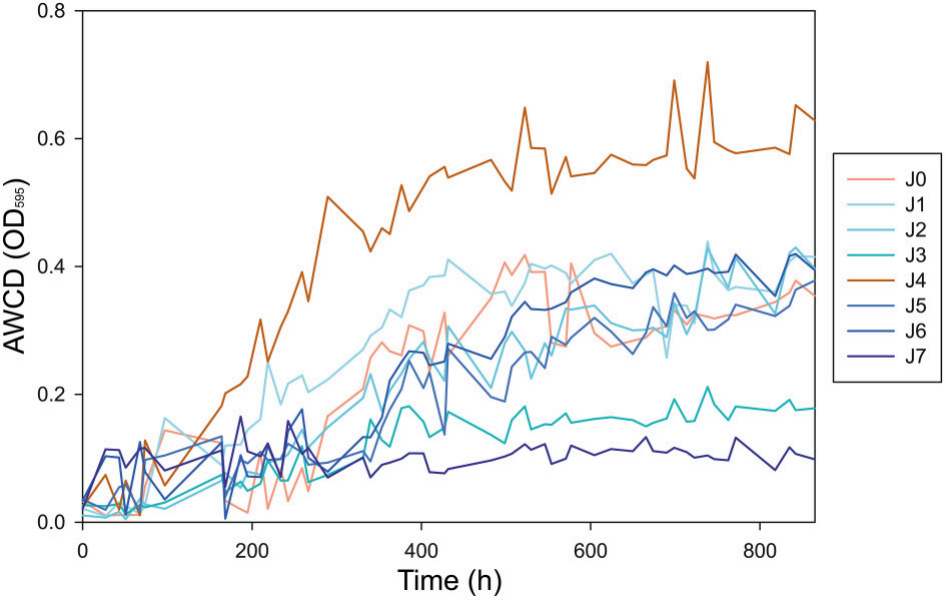
**Increase of the AWCD OD_**(595)**_ during incubation over 865 h (36 days) at 4°C**. Peak metabolic activity was observed after 739 h (31 days) of incubation.

We analyzed the metabolic rates of all samples on different carbon substrates. Average Well Color Development of OD_(595)_ (AWCD) began to increase significantly in most samples after 120 h of incubation at 4°C (Figure 10). The AWCD increased constantly until hour 432 (day 18) of incubation, at which point most samples reached a plateau. The strongest activity was observed in J4, which reached an AWCD value of 0.72 after 739 h (day 31) and had an AWCD significantly higher than the other samples (*P* < 0.05). AWCD in J0, J1, J2, J5, and J6 achieved steady state values between 0.37 and 0.44 after 739 h. Samples J3 and J7 showed lower metabolic activity than the others, with AWCD values of 0.21 and 0.10, respectively (Figure 10). Negative control with CHCl_3_−fumigated samples showed no activity in all samples (data not shown).

**Figure 11:**
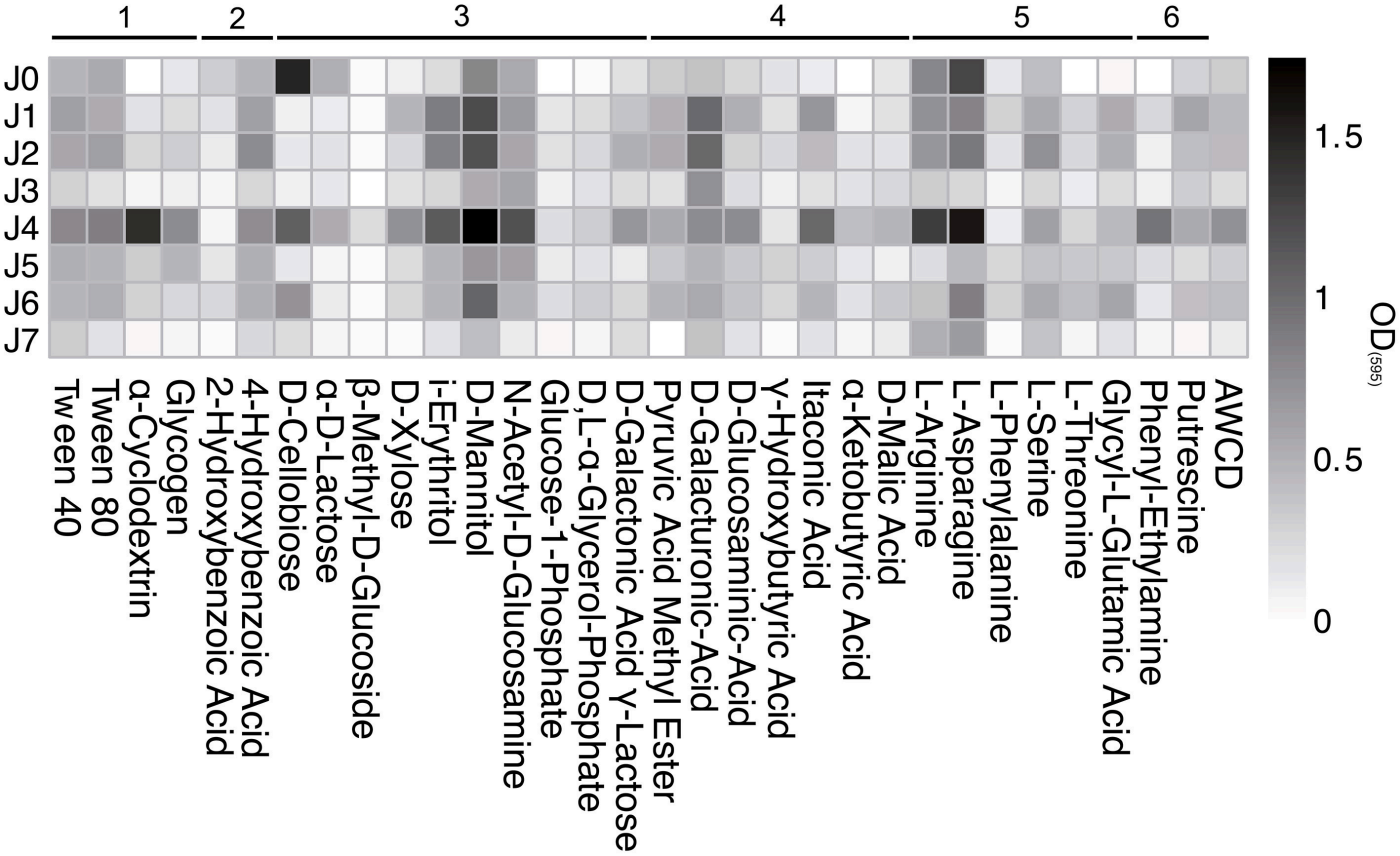
**Biolog EcoPlate™ after 739 h (31 days) at 4°C**. Metabolization assay of different substrates: 1, polymers; 2, phenolic compounds; 3, carbohydrates; 4, carboxylic acids; 5, amino acids; 6, amines. AWCD, Average Well Color Development of OD_(595)_.

The polymers Tween 40 and 80 showed a strong increase from the start of incubation and plateaued at high values in all samples (Figure 11). The polymers α-Cyclodextrin and Glycogen were metabolized only in J4 and J5. Of the ten carbohydrate substrates tested, D-Mannitol was generally metabolized the best, followed by i-Erythritol, N-Acetyl-D-Glucosamine and D-Xylose.

D-cellobiose and α-d-lactose were strongly metabolized by bacteria in both SD-layers, but only slightly in the CS-layers. The consumption of D-Galactonic Acid γ-Lactose was observed only in J4, and D-Galacturonic Acid was the only carboxylic acid metabolized above AWCD in all samples. The utilization of Pyruvic Acid Methyl Ester, D-Glucosaminic Acid and Itaconic Acid was generally low, but highest in J4. The amino acids L-Asparagine and L-Arginine showed the strongest consumption rates compared to all other carbon sources, with the highest value in the SD-layers.

## Discussion

### Deposition and trajectory

Generally, SD emission is low in winter and SDEs that travel toward Europe are rare (Shao et al., 2011). Only 29% of all SDEs last longer than 24 h; evidence of such events is most commonly found at the Jungfraujoch between March and June and between October and November (Collaud Coen et al., 2004; Flentje et al., 2015). The winter SDE of February 2014 was therefore exceptional in terms of both season and duration. Since the Jungfraujoch is situated above the planetary boundary layer in winter, aerosols are not exposed to anthropogenic contamination after their deposition on the snow. However, it is impossible to exclude anthropogenic contamination during the transport. Moreover, the SD particles from the winter SDE were deposited as a thin layer and covered by fresh snow 3 days after the SDE. The SD-layer was therefore preserved under freezing conditions until the sampling. Based on these observations, we could link the well-preserved J4 SD-layer to the winter SDE of February 2014. The less well-defined SD-layer J0, affected by freeze-thaw cycles, corresponded to the spring SDE deposited in May 2014.

Backward trajectories were used to project the main potential source region of the SDEs (Figure 1). The trajectory analysis suggests that the winter SDE (J4) originated in central-southern Algeria and, to a lesser extent, in the neighboring countries. The SD particles uplifted in the desert remained in the atmosphere until their deposition on the Jungfraujoch. The trajectory of the spring SDE (J0) indicates that its potential source region was northwestern Algeria. This is in agreement with the observation of Collaud Coen et al. (2004) that 90% of the SDEs detected in Switzerland potentially originate in Algeria, although the source can vary slightly between seasons. Although the NOAA HYSPLIT trajectories represent interpretations from well-established models for atmospheric processes, the results of the model have to be treated with caution as they give only approximate information of the dust origin.

### Geochemical and mineralogical characteristics

The SD-layers were significantly enriched in dust particles as compared to the CS-layers, and particularly enriched in undefined mixed-phase particles. These mixed-phase particles were absent in the surrounding bedrock, indicating an allochthonous origin of those particles. The average bulk composition of SD particles was dominated by SiO_2_ and Al_2_O_3_ followed by other commonly reported oxides (Collaud Coen et al., 2004; Krueger et al., 2004; Goudie and Middleton, 2006). Although the northwestern Saharan Atlas region is characterized by carbonates (Moreno et al., 2006; Sodemann et al., 2006), no enrichment of CaO was observed in J0, which originated from that region according to the backward trajectory.

SD particles are generally dominated by silica (quartz and diatoms), clay minerals (illite, kaolinite, montmorillonite or palygorskite), feldspars, carbonates (calcite, dolomite) and evaporitic minerals (gypsum, halite) (Evans et al., 2004; Moreno et al., 2006) and are of similar composition to SD aerosols collected at the Jungfraujoch (Schwikowski et al., 1995; Collaud Coen et al., 2004). The dissolution of evaporitic minerals could explain the significantly higher conductivity, lower pH and lower snow density observed in the SD-layers as compared to the CS-layers.

The illite/kaolinite (I/K) ratio has been proposed as an effective mineralogical signature for discriminating between SD source regions (Caquineau et al., 2002; Scheuvens et al., 2013). Dust from the western and northern Sahara exhibit the highest amounts of illite, whereas kaolinite dominates soils of the southern Sahara and Sahel (Caquineau et al., 2002). The I/K ratios observed in the SD-layers support the hypothesis that J0 originated in northwestern Algeria and J4 in central-southern Algeria.

Diatoms were detected only in J4. This suggests that the dust particles in J4 originated further south than those in J0. In fact, detrital diatoms originate from the Bodélé Depression, which was exposed to the wind after the retreat of the Mega-Lake Chad (Romero et al., 1999). Together, clay particles and diatom fragments can form armored clay-diatom agglomerations that offer bacteria dwelling spaces protected from UV radiation (Chappell et al., 2008; Favet et al., 2013).

Despite the importance of the mineralogical signature of the source region, the long-range transport of aerosols inevitably leads to fractionation processes that alter the size and mineralogical composition of the aerosol particles during their transport to the Alps (Claquin et al., 1999; Moreno et al., 2006). Fractionation processes and mixing of dust particles during transport further hamper a unequivocal identification of the source region based on the geochemical or mineralogical composition of the aerosols (Sodemann et al., 2006). In addition, the mineralogical signature of aerosols reaching the Jungfraujoch can vary substantially from SDE to SDE depending on the season (Sodemann et al., 2006).

### Microbiology

As expected, bacterial richness and concentration, in terms of 16S rRNA gene copy per ml snowmelt, were higher in the SD-layers as compared to the CS-layers. Bacterial concentration was positively correlated with the higher dust concentration, which is in agreement with previous studies (Segawa et al., 2005). All diversity indices and evenness presented higher values in the SD-layers as compared to the CS-layers with significant differences for the Inverse Simpson and Shannon indices. The Shannon diversity index value of the SD-layers at Jungfraujoch was similar to that observed in atmospheric SD collected in Spain (Sánchez De La Campa et al., 2013), which corresponds to the lower limit of diversity commonly observed in soils (Fahlgren et al., 2010).

Interestingly, the ratio of DNA quantity to 16S rRNA gene copy was higher in J0 than in J4. This may be due to a stronger presence of biological particles other than bacteria in J0, such as eukaryotes (e.g., fungi, protists, pollen). Pollen in particular is more abundant in spring, the season in which J0 was deposited. In winter, the season in which J4 was deposited, decaying cellular matter prevails (Jaenicke, 2005; Be et al., 2015).

### Common airborne bacteria and pathogens

The gram-negative *Proteobacteria* accounted for 70–80% of the bacterial community, a value similar to that found for aerosols from the Sahara Desert collected in Spain (Sánchez De La Campa et al., 2013; Barberán et al., 2014). However, only a few genera were dominant in the CS-layers and SD-layers. Both SD-layers contained high abundances of *Betaproteobacteria Oxalobacteriaceae* and *Comamonadaceae*, in agreement with observations of SD deposited on snow in the Central Pyrenees (Spain) (Hervas et al., 2009; Hervas and Casamayor, 2009).

The psychrophilic *Janthinobacterium* was present in varying abundances throughout the snowpack, confirming it as a regular member of the airborne bacterial community (Fahlgren et al., 2010). In fact, it was also found in cold environments such as snow (Chuvochina et al., 2011a), glaciers (Kim et al., 2012), alpine lakes (Peter et al., 2014) and attached to granitic rock particles in soils of the Damma glacier forefield (Switzerland) (Frey et al., 2010). *Janthinobacterium* produces the indigo-purple pigment violacein (Pantanella et al., 2007), which is an antifungal metabolite against amphibian skin pathogens and is enhanced by the carbohydrate mannitol (Brucker et al., 2008). Previous studies have shown that mannitol is suitable as chemical tracer for fungal spores in atmospheric aerosols (Elbert et al., 2007). All of our samples showed strong metabolic activity when combined with D-mannitol, most notably SD-layer J4. *Janthinobacterium* also produces weathering-associated compounds such as oxalic acids and cyanide, which dissolve granite and lead to a reduction in the pH of the soil (Frey et al., 2010). Similar granite dissolution activity has been observed for the psychrotrophic genera *Variovorax* and *Polaromonas* (Frey et al., 2010), members of the family *Comamonadaceae*, which were detected in significant abundances exclusively in the SD-layers.

*Pseudomonas*, which was the most abundant gammaproteobacterium in the CS-layers and J4, has been shown to be a persistent member of the airborne community throughout the year (Fahlgren et al., 2010), and to be involved in cloud condensation processes (Amato et al., 2007a) and ice nucleation at temperatures close to 0°C (Möhler et al., 2007; Bowers et al., 2009).

Pathogens such as *Neisseria meningitidis, Streptococcus pneumonia* and *Haemophilus influenza*e are present in the “meningitis belt” of sub-Saharan Africa and known to cause meningitis. In our study, six OTUs potentially belonging to these pathogens were detected in all samples, although in very low abundances. These include two members of the family *Neisseriaceae*, one member of an unknown genus and one *Neisseria*; three members of the genus *Streptococcus*, one member belonging to the species *infantis*, and one *Haemophilus parainfluenzae*.

### Bacteria specific to Saharan dust

We observed strong similarities between the bacterial community structures of the J0 and J4 SD-layers. The genera *Hymenobacter* (*Bacteroidetes*) and *Comamonodaceae* (*Betaproteobacteria*) were exclusively present in considerable abundances in the SD-layers. Other phylotypes adapted to harsh conditions, including *Gemmatimonadetes, Deinococcus-Thermus, Chloroflexi* and the deltaproteobacterium *Myxococcales*, were also found mostly in the SD-layers. Other phylotypes that were particularly abundant in the SD-layers, such as the two *Bacteroidetes* families *Flavobacteraceae* and *Cytophagaceae*, were also detected in the CS-layers.

*Gemmatimonadetes* and *Chloroflexi* have been found in air masses passing over the Sahara Desert, but are absent in air masses passing over Southern Europe (Katra et al., 2014). *Gemmatimonadetes* have been observed in nearly all soil types (Fierer et al., 2012), but are predominant in soils from hyper-arid environments with very low biomass, such as the Atacama Desert (Drees et al., 2006; Crits-Christoph et al., 2013), the Sahara Desert (Favet et al., 2013), the Tatouine Desert (Chanal et al., 2006) and McKelvey Valley (Antarctica) (Pointing et al., 2010). In fact, their presence is inversely correlated to soil moisture (Debruyn et al., 2011), indicating a tolerance for desiccation and oligotrophic conditions related to very slow cell growth (Zhang, 2003). *Gemmatimonadetes* have recently been found to be dominant in soils of recently deglaciated and unvegetated alpine glacier forefields (Bajerski and Wagner, 2013; Rime et al., 2015). These bacteria may originate in the Sahara Desert and be transported to the Alps following SDEs, where they find suitable environments to proliferate. Rare orange pigments (carotenoids) that function as antioxidants may help *Gemmatimonadetes* protect themselves against DNA damage caused by UV radiation (Tong and Lighthart, 1997; Hanada and Sekiguchi, 2014).

The Gram negative genus *Deinococcus* also showed a higher species richness in the SD-layers compared to the CS-layers. *Deinococcus spp*. are pink to red pigmented bacteria that are adapted to desiccation and radiation (De Groot et al., 2005; Callegan et al., 2008). They have been isolated from a wide range of arid environments including Antarctica (Hirsch et al., 2004) the Gobi Desert (Yuan et al., 2009), the Sonoran Desert (Rainey et al., 2005), the Sahara Desert (De Groot et al., 2005; Favet et al., 2013) and the Tataouine Desert (Chanal et al., 2006), as well as from alpine soils (Callegan et al., 2008). *Deinococcus* have been reported in various studies of aerosols associated with bacteria originating from the Gobi Desert (Jeon et al., 2011; Yamaguchi et al., 2014) and from the Sahara Desert (Chuvochina et al., 2011a). Therefore, both *Gemmatimonadetes* and *Deinococcus-Thermus* could be considered bio-indicators for SDEs.

The two *Bacteroidetes* families *Cytophagaceae* and *Flavobacteriaceae* are also abundant in the SD-layers and have been found in soils from the Sahara Desert in Chad (Favet et al., 2013) and Tunisia (Chuvochina et al., 2011b). In fact, *Bacteroidetes* prefer desert to non-desert soils (Fierer et al., 2012). Moreover, *Bacteroidetes* were also highly abundant in aerosols collected over Japan (Yamaguchi et al., 2014) and over eastern Australia (De Deckker et al., 2014). *Bacteroidetes* adhere preferentially to smaller particle sizes of Saharan aerosols (Polymenakou et al., 2008), which might contribute to their long-distance propagation. The most abundant phylotypes detected in the SD-layers were the pigmented and psychrotolerant *Hymenobacter* (*Cytophagaceae*) and *Flavobacterium*. *Hymenobacter* (*Flavobacteraceae*) was also detected in snow from the Guoqu and Zadang glaciers on the Tibetan Plateau covered with dust from Chinese deserts (Liu et al., 2009), in SD-bearing snow on Mont Blanc (Chuvochina et al., 2011b), from desert soil in Chad (Favet et al., 2013) and over the Caribbean during a SDE (Griffin et al., 2003).

### Survival of airborne bacteria

There is solid evidence in the literature that at least a fraction of airborne microorganism is viable (Deleon-Rodriguez et al., 2013). Based on microscopy observations, 60–100% of the bacteria appeared viable in samples from the upper troposphere (Deleon-Rodriguez et al., 2013), and were metabolically active in clouds (Hill et al., 2007). However, microscopy-based values might be overestimated if inorganic particles are stained and erroneously counted as cells (Deleon-Rodriguez et al., 2013). Moreover, limitations of traditional culture-based techniques prevent researchers from precisely assessing viability of airborne bacteria (Amato et al., 2015; Behzad et al., 2015). Biolog® incubations are partially also subject to these limitations although they are advanced cultivation-based techniques that permit the detection of metabolic activity using various carbon substrates.

Using Biolog® incubations, we were able to quantify the mean metabolic activity of the bacterial communities in each snow layer and to hence determine their viability. Bacterial communities taken from the SD-layers showed no lag phase in the Biolog® incubations suggesting that the bacteria were viable (Figure 10).

Previous studies have shown that specific airborne bacteria metabolize certain carbon substrates such as glucose (Dimmick et al., 1975) and carboxylic acids (i.e., formate, acetate, succinate, and lactate) (Amato et al., 2005). In this study, metabolic activity was higher on substrates such as the sugar L-mannitol and the amino acid L-asparagine. Although bacterial activity was higher in certain substrates than in others, it was not possible to relate individual species of airborne bacteria to specific carbon substrates.

## Conclusions

Airborne bacteria are exposed to harsh conditions during transport in the upper troposphere, including desiccation stress, UV radiation, low temperatures and oligotrophic conditions. Bacteria possessing adaptive strategies (i.e., sporulation and pigmentation) were expected to be highly abundant in the SD-layers at the Jungfraujoch. In contrast to the findings of other studies (Polymenakou et al., 2008; Chuvochina et al., 2011a), however, we observed neither higher species richness nor higher abundances of sporulating bacteria in the SD-layers as compared to the CS-layers, which is in agreement with Barberán et al. (2014). Instead, we found a higher species richness of pigment-producing bacteria that are adapted to cope with UV radiation and desiccation stress such as *Gemmatimonadetes, Deinococcus-Thermus, Chloroflexi, and Deltaproteobacteria Myxococcales*.

## Author contributions

MM, AL, and JZ designed the study and did the fieldwork. MM performed the experiments, analyzed the data and wrote the study. AL and JZ contributed to the writing.

### Conflict of interest statement

The authors declare that the research was conducted in the absence of any commercial or financial relationships that could be construed as a potential conflict of interest.
