# Supplementary material for: Bacterial Composition and Survival on Sahara Dust Particles Transported to the European Alps
Source: Front Microbiol. 2015 Dec 22;6:1454. doi: 10.3389/fmicb.2015.01454 (PMC4686684; doi:10.3389/fmicb.2015.01454)
Supplement: Supplementary file 1 [file DataSheet1.PDF]

Supplementary Material

Bacterial composition and survival on Sahara dust particles transported to the European Alps

Meola Marco[1], Lazzaro Anna[1], Zeyer Josef[1]*

*** Correspondence:** Josef Zeyer, Environmental Microbiology, Institute of Biogeochemistry and Pollutant Dynamics, Environmental Systems Science, Swiss Federal Institute of Technology (ETH Zurich), ETH-Zentrum CHN, Universitätstrasse 16, CH-8092 Zurich, Switzerland.

E-mail: josef.zeyer@env.ethz.ch

Supplementary Figures and Tables

Supplementary Figures

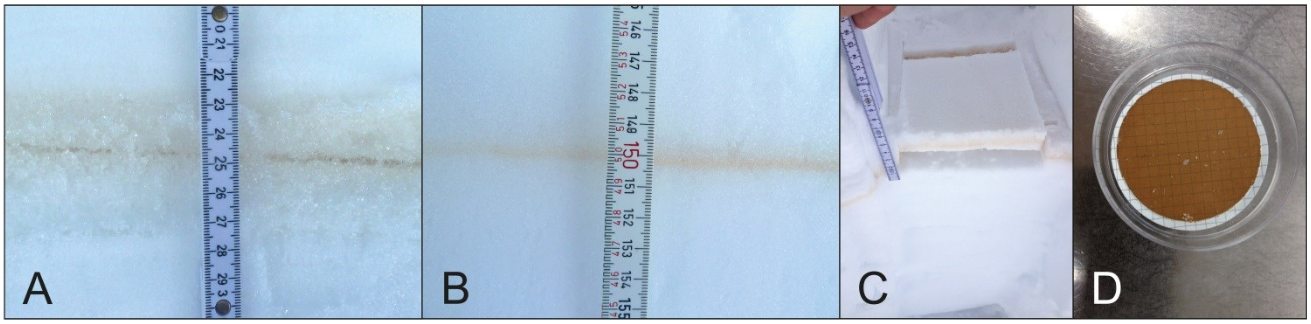

Supplementary Figure 1: A) The SD-layer J0 in the snowpack. B) The SD-layer J4 in the snowpack. C) Sampling of the SD-layer J4. D) Filter covered with dust particles after filtration of J4.

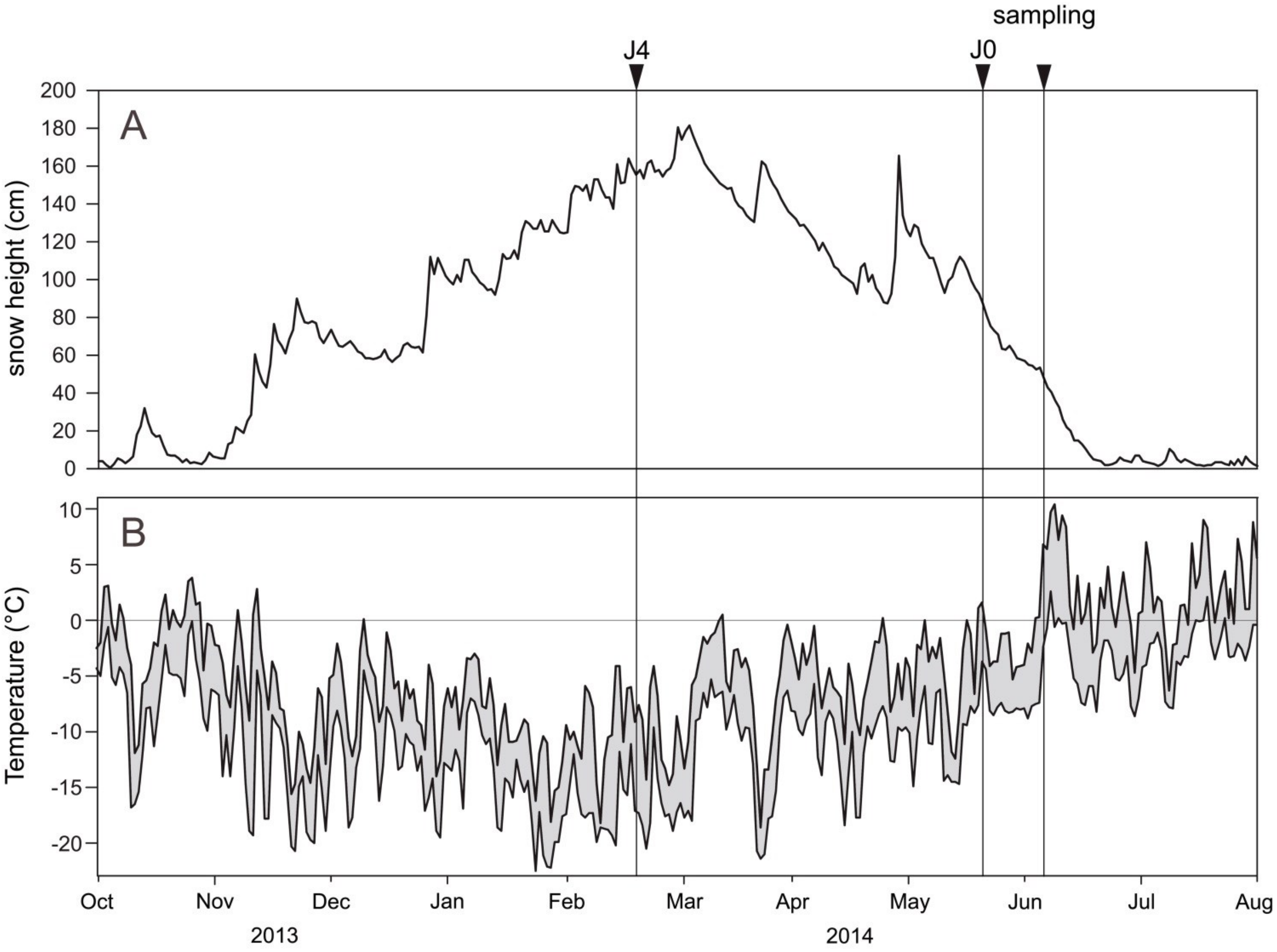

Supplementary Figure 2: A) Average snow height from the nearest meteorological stations to the Jungfraujoch, Männlichen and Eggishorn. Please note that snow accumulation after March is different at Jungfraujoch due to its higher elevation. Moreover, the ground at Jungfraujoch is composed of glacier ice, whereas in Männlichen and Eggishorn it is soil. In contrast to the meteorological stations located at 2343 m a.s.l. and 2927 m a.s.l., respectively, no snowmelt occurred at Jungfraujoch before 6 June 2014. B) Day- and night-time air temperature data from the Jungfraujoch meteorological station. SDE 18-19 February 2014 = J4; SDE 22 May 2014 = J0.

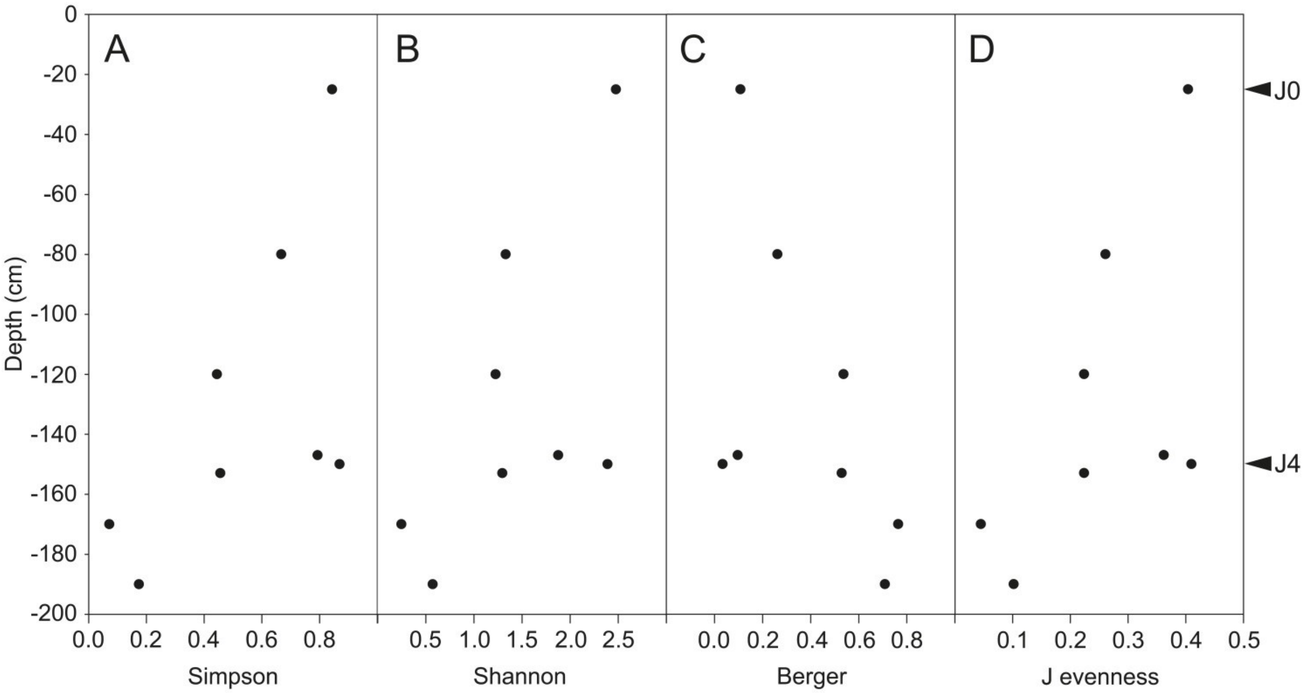

Supplementary Figure 3: Diversity indices Simpson (A), Shannon (B), Berger (C) and J' evenness (D) on bacterial communities of merged field replicates, therefore no standard deviations are shown.

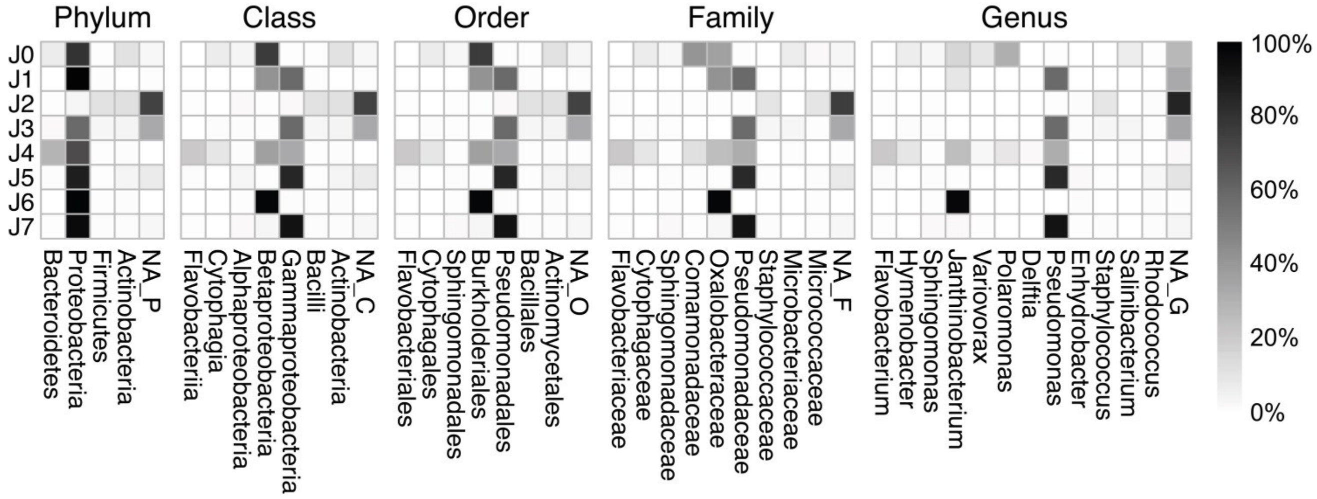

Supplementary Figure 4: Normalized abundances of the dominant members (>1%) at different taxonomic levels.

Supplementary Tables

Name	*Overhang*	*Insert*	*Primer*
B341F_fs0	*TCGTCGGCAGCGTCAGATGTGTATAAGAGACAG*		CCTACGGGNGGCWGCAG
B341F_fs1	*TCGTCGGCAGCGTCAGATGTGTATAAGAGACAG*	***N***	CCTACGGGNGGCWGCAG
B341F_fs2	*TCGTCGGCAGCGTCAGATGTGTATAAGAGACAG*	***NN***	CCTACGGGNGGCWGCAG
B341F_fs3	*TCGTCGGCAGCGTCAGATGTGTATAAGAGACAG*	***NNN***	CCTACGGGNGGCWGCAG
B805R_fs0	*GTCTCGTGGGCTCGGAGATGTGTATAAGAGACAG*		GACTACHVGGGTATCTAATCC
B805R_fs1	*GTCTCGTGGGCTCGGAGATGTGTATAAGAGACAG*	***N***	GACTACHVGGGTATCTAATCC
B805R_fs2	*GTCTCGTGGGCTCGGAGATGTGTATAAGAGACAG*	***NN***	GACTACHVGGGTATCTAATCC
B805R_fs3	*GTCTCGTGGGCTCGGAGATGTGTATAAGAGACAG*	***NNN***	GACTACHVGGGTATCTAATCC

Supplementary Table 1: Forward primer B341F and reversed primer B805R with overhang (*italic)* and an insertion of zero to three nucleotides between the overhang and the primer sequence (**bold**).

Step	Process	Software	Reference
1	quality control	FastQC v0.10.1	www.bioinformatics.bbsrc.ac.uk/projects/fastqc
2	merging	FLASH v1.4.2	(Magoc and Salzberg, 2011)
3	primer trimming	cutadapt v1.4.2	(DOI: http://dx.doi.org/10.14806/ej.17.1.200)
4	phylotype clustering	Usearch v7.0. 1090_i86linux64	(Edgar, 2010)
5	BLAST and taxonomic assignment	Greengenes database v13_5.	

Supplementary Table 2: Step 3: overlap: full length, error rate: 0. Step 4: min. quality mean: 25, no ambiguous nucleotides, length range: 450-550 bp, GC range: 20-80%. Step 5: abundance sorting: 2, id=97%, chimera filtering.

Sample	*SiO_2*	*Al_2O_3*	*Fe_2O_3*	*K_2O*	*MgO*	*CaO*	*Na_2O*	*P_2O_5*	*TiO_2*	*SO_3*	*MnO*	*Cr_2O_3*	*V_2O_5*	*ZrO_2*
							[wt%]							
J0	60.0	21.5	9.5	3.6	3.6	0.6	0.4	0.4	0.2	0.2	0.0	0.0	0.0	0.0
J1	50.7	12.8	6.0	2.1	3.1	2.0	2.4	0.0	0.0	3.6	0.0	17.1	0.2	0.0
J2	54.7	20.1	10.1	3.2	2.8	0.2	0.3	0.8	1.0	1.9	0.0	4.6	0.0	0.2
J3	61.9	13.4	5.5	3.8	1.5	1.2	0.4	3.0	0.5	3.4	0.0	5.2	0.0	0.0
J4	58.2	23.7	10.7	2.2	2.5	0.8	0.4	0.1	0.9	0.3	0.1	0.2	0.0	0.0
J5	48.8	19.7	11.6	3.6	3.1	2.7	0.3	1.4	2.3	1.7	0.0	4.9	0.0	0.0
J6	53.7	24.1	8.7	2.9	2.1	2.9	0.5	0.0	2.1	0.9	0.0	2.0	0.0	0.0
J7	58.4	21.6	10.8	2.9	3.1	0.3	0.3	0.4	1.3	0.9	0.0	0.0	0.0	0.0
Rock1	56.6	26.1	6.5	7.1	2.4	0.0	1.1	0.0	0.3	0.0	0.0	0.0	0.0	0.0
Rock2	70.9	17.8	1.9	6.5	0.7	1.0	0.1	0.8	0.2	0.0	0.0	0.0	0.0	0.0
Rock3	9.1	5.5	9.3	0.6	1.5	72.9	0.0	0.5	0.1	0.3	0.1	0.0	0.0	0.0

Supplementary Table 3: Geochemical composition of the dust particles in the snow samples (J0-J7) and the surrounding bedrocks (Rock1, Rock2 and Rock3).

	J0	J1	J2	J3	J4	J5	J6	J7	CS-Layers
Acidobacteria	0.05	0.00	0.04	0.00	0.00	0.27	0.00	0.01	0.05
Actinobacteria	10.43	0.06	11.36	3.79	0.58	3.52	0.89	0.86	3.42
Bacteroidetes	6.48	0.04	0.18	1.21	29.24	0.78	0.03	0.28	0.42
Chloroflexi	0.16	0.00	0.00	0.02	0.10	0.06	0.00	0.01	0.02
Cyanobacteria	0.27	0.02	1.28	0.18	0.01	0.55	0.07	0.03	0.36
Deinococcus-Thermus	0.23	0.00	0.01	0.00	0.01	0.01	0.00	0.00	0.00
Elusimicrobia	0.00	0.00	0.00	0.00	0.00	0.00	0.00	0.00	0.00
FBP	0.00	0.00	0.00	0.00	0.00	0.00	0.00	0.00	0.00
Firmicutes	0.30	0.12	10.52	2.95	0.16	0.33	0.11	0.71	2.46
Gemmatimonadetes	0.06	0.00	0.00	0.00	0.02	0.00	0.00	0.00	0.00
OD1	0.00	0.00	0.01	0.00	0.00	0.00	0.00	0.00	0.00
Planctomycetes	0.00	0.00	0.00	0.00	0.00	0.02	0.00	0.00	0.00
Proteobacteria	79.66	99.59	2.87	58.87	69.79	87.40	98.88	95.98	73.93
WPS-2	0.00	0.00	0.00	0.00	0.00	0.02	0.00	0.00	0.00
NA	2.35	0.16	73.73	32.97	0.08	7.03	0.02	2.12	19.34

Supplementary Table 4: Presence and abundance of sample-specific phylotypes. Distribution of phyla OTUs in terms of abundance and uniqueness among J0, J4 and CS-layers. OTUs (#) = number of OTUs; Abund. (%) = abundance in %; Unique (#) = number of OTUs unique in that sample; Unique (%) = rel. importance among all unique OTUs; Unique Abund. (%) = rel. importance among all OTUs.

Phylum	All (539)		J0 (459)					J4 (339)					clean snow (427)				
	OTUs	*Abund.*	*OTUs*	*Abund.*	*Unique*	*Unique*	*Unique Abund.*	*OTUs*	*Abund.*	*Unique*	*Unique*	*Unique Abund.*	*OTUs*	*Abund.*	*Unique*	*Unique*	*Unique Abund.*
	#	%	#	%	#	%	%	#	%	#	%	%	#	%	#	%	%
Acidobacteria	15	0.04	14	0.05	0	0.00	0.00	9	0.00	0	0.00	0.00	13	0.05	1	1.72	6.67
Actinobacteria	117	4.81	101	10.43	2	5.26	1.71	84	0.58	0	0.00	0.00	101	3.42	12	20.69	10.26
Bacteroidetes	64	12.05	57	6.48	14	36.84	21.88	32	29.24	2	33.33	3.13	38	0.42	5	8.62	7.81
Chloroflexi	53	0.09	46	0.16	4	10.53	7.55	43	0.10	3	50.00	5.66	30	0.02	3	5.17	5.66
Cyanobacteria	22	0.21	16	0.27	2	5.26	9.09	11	0.01	0	0.00	0.00	19	0.36	3	5.17	13.64
Deinococcus-Thermus	15	0.08	14	0.23	1	2.63	6.67	10	0.01	1	16.67	6.67	11	0.00	0	0.00	0.00
Elusimicrobia	1	0.00	0	0.00	0	0.00	0.00	0	0.00	0	0.00	0.00	1	0.00	1	1.72	100.00
FBP	1	0.00	1	0.00	1	2.63	100.00	0	0.00	0	0.00	0.00	0	0.00	0	0.00	0.00
Firmicutes	54	0.97	39	0.30	3	7.89	5.56	27	0.16	0	0.00	0.00	49	2.46	11	18.97	20.37
Gemmatimonadetes	16	0.03	16	0.06	2	5.26	12.50	13	0.02	0	0.00	0.00	4	0.00	0	0.00	0.00
OD1	1	0.00	1	0.00	0	0.00	0.00	0	0.00	0	0.00	0.00	1	0.00	0	0.00	0.00
Planctomycetes	5	0.00	2	0.00	0	0.00	0.00	1	0.00	0	0.00	0.00	5	0.00	2	3.45	40.00
Proteobacteria	168	74.46	148	79.66	9	23.68	5.36	105	69.79	0	0.00	0.00	148	73.93	18	31.03	10.71
WPS-2	1	0.00	1	0.00	0	0.00	0.00	0	0.00	0	0.00	0.00	1	0.00	0	0.00	0.00
NA	6	7.26	3	2.35	0	0.00	0.00	4	0.08	0	0.00	0.00	6	19.34	2	3.45	33.33
Total	539	100.00	459	100.00	38	100.00	7.05	339	100.00	6	100.00	1.11	427	100.00	58	100.00	10.76

Supplementary Table 5: Abundance of all phyla in all snow layers and average abundance in all CS-layers. Values in % normalized relative abundance.
